# Targeting envelope proteins of poxviruses to repurpose phytochemicals against monkeypox: An *in silico* investigation

**DOI:** 10.3389/fmicb.2022.1073419

**Published:** 2023-01-05

**Authors:** Pallavi Gulati, Jatin Chadha, Kusum Harjai, Sandeepa Singh

**Affiliations:** ^1^Department of Microbiology, University of Delhi, New Delhi, India; ^2^Department of Microbiology, Panjab University, Chandigarh, India; ^3^Department of Botany, Maitreyi College, University of Delhi, New Delhi, India

**Keywords:** poxviruses, monkeypox virus, envelope proteins, drug repurposing, bioactive phytochemicals, molecular docking, molecular simulation

## Abstract

The monkeypox virus (MPXV) has become a major threat due to the increasing global caseload and the ongoing multi-country outbreak in non-endemic territories. Due to limited research in this avenue and the lack of intervention strategies, the present study was aimed to virtually screen bioactive phytochemicals against envelope proteins of MPXV *via* rigorous computational approaches. Molecular docking, molecular dynamic (MD) simulations, and MM/PBSA analysis were used to investigate the binding affinity of 12 phytochemicals against three envelope proteins of MPXV, *viz*., D13, A26, and H3. Silibinin, oleanolic acid, and ursolic acid were computationally identified as potential phytochemicals that showed strong binding affinity toward all the tested structural proteins of MPXV through molecular docking. The stability of the docked complexes was also confirmed by MD simulations and MM/PBSA calculations. Results from the iMODS server also complemented the findings from molecular docking and MD simulations. ADME analysis also computationally confirmed the drug-like properties of the phytochemicals, thereby asserting their suitability for consumption. Hence, this study envisions the candidature of bioactive phytochemicals as promising inhibitors against the envelope proteins of the MPXV, serving as template molecules that could further be experimentally evaluated for their efficacy against monkeypox.

## 1. Introduction

In recent years, the world has witnessed the resurgence of several viral infections, including the Nipah virus, influenza virus (H1N1, H5N1), Ebola virus, Zika virus, and the recent coronavirus outbreaks (MERS-CoV and SARS-CoV-2; Mourya et al., 2019; Chadha et al., 2022c). The latest addition to this list is the monkeypox virus (MPXV), one of the four human pathogenic orthopoxvirus species, grouped along with variola, cowpox, and vaccinia virus (Lam et al., 2022). Monkeypox (MPX) is an emerging zoonotic disease caused by the MPXV, which was first discovered in 1958 following local outbreaks of a smallpox-like disease in monkey colonies maintained at research facilities in Africa and Denmark (Chadha et al., 2022b). However, the first clinical case of MPX (in humans) was reported in 1970 in the Equatorial province of the Democratic Republic of Congo (DRC; Marennikova et al., 1972). Since then, sporadic MPX outbreaks have been reported across several African countries, particularly the DRC, Republic of The Congo, Central African Republic, Nigeria, Liberia, Ghana, and Cameroon (Bunge et al., 2022). The MPXV has been endemic to sub-Saharan countries for decades, but in mid-2022 (amid the COVID-19 pandemic), an unexpected surge in MPX infections was recorded globally (WHO, 2022). Multi-country outbreak of MPX was reported across several non-endemic countries, raising concern among clinicians and epidemiologists (Chadha et al., 2022b). With the increasing global caseload, the World Health Organization also declared the ongoing multi-country outbreak of MPX as a Public Health Emergency of International Concern (PHEIC) on 23rd July 2022.

The MPXV is an enveloped virus with a dsDNA genome of nearly 190 kbp and harbors a dumbbell-shaped core with lateral bodies (Kugelman et al., 2014). The virus is known to infect various mammalian species, including primates, but its natural animal reservoir remains unknown. However, some investigations suggest that rodents are the primary reservoir of the MPXV (Reynolds et al., 2012). To date, two distinguishable clades of MPXV have been identified: West African and Central African (Congo Basin). The latter is highly transmissible among humans and exhibits potentially high virulence and case fatality rate (~ 11%) (Sklenovská and Van Ranst, 2018). MPX transmission has been strongly associated with animal bites, skin abrasions, prolonged or direct contact with bodily fluids of infected animals or humans, respiratory droplets, and contaminated inanimate objects (Ligon, 2004). However, the transmission of presently-circulating MPXV strains through sexual routes is still debated (Chadha et al., 2022b). The incubation period of MPXV ranges from 3 to 14 days, and the clinical manifestations are seldom mistaken for other viral infections like herpes simplex virus and chickenpox. Symptoms of MPX are similar to that of smallpox, including fever accompanied by blisters, muscle pain, rashes, chills, and respiratory illness (Titanji et al., 2022). The present-day treatment and clinical management of MPX involve the administration of repurposed antiviral drugs (cidofovir, brincidofovir, and tecovirimat) and preventive vaccines (ACAM2000 and JYNNEOS; Chadha et al., 2022b). Moreover, around 50 single-nucleotide polymorphisms have been identified in the genome of 2022 MPXV, which remained undetected in the previously circulating MPXV strains between 2018 and 2019 (Isidro et al., 2022). This increases the gravity of the ongoing multi-country outbreak, pointing toward the “accelerated evolution” of MPXV. Hence, there is a pressing need to develop novel therapeutics against the MPXV. In this direction, drug repurposing proves to be an effective alternative since it bypasses the need for drug development, thereby reducing the time frame for drug discovery (Gulati et al., 2021).

**Figure 1:**
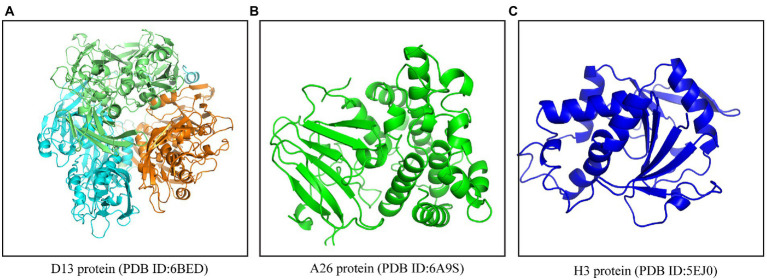
Crystal structures of envelope proteins of MPXV. **(A)** D13 protein trimer (PDB ID: 6BED), **(B)** A26 protein (PDB ID: 6A9S), **(C)** H3 protein (PDB ID: 5EJ0). The structures were retrieved from the PDB online server (www.rcsb.org).

Due to their natural existence, easy availability, extensive antimicrobial, antiviral, antivirulence, and pharmacological properties, the application of plant-derived products and bioactive phytochemicals in modern medicine has gained momentum among researchers (Chadha et al., 2021, 2022a). Numerous *in silico* and pharmacoinformatics-based reports have predicted the effectiveness of phytochemicals and other drugs against SARS-CoV-2, Ebola, and Nipah virus (Kwofie et al., 2019; Pathania et al., 2019; Choudhury et al., 2021; Kumar A. et al., 2021). On similar lines, we employed a comprehensive *in silico* approach to identify phytochemicals that could potentially act as inhibitors against poxviruses for repurposing them against the MPXV. In a recent study, Lam et al. demonstrated a high sequence similarity among the structural proteins of viruses belonging to *Poxviridae*, especially between the MPXV and vaccinia virus (Lam et al., 2022). Contingent to this, we utilized the structural proteins of the vaccinia virus, which is a close relative of MPXV, as the drug target(s). The first step toward any successful viral infection is cellular entry, which is facilitated by the envelope proteins that are involved in the attachment of the virus to the host cell. Hence, drugs that target the envelope proteins could potentially inhibit viral infection at an early stage. Considering this fact, the phytochemicals included in this study were targeted against the three structural proteins (envelope) of poxviruses *viz*., D13, A26, and H3 (Figure 1) using molecular docking coupled with dynamic simulations and also evaluated for their pharmacokinetic properties and druglikeliness.

## 2. Materials and methods

### 2.1. ADME analysis

The ligands under consideration (phytochemicals) were initially screened to eliminate the compounds violating Lipinski’s rule of five (Benet et al., 2016). The molecular and physicochemical properties of the phytochemicals were investigated using the SwissADME tool (Daina et al., 2017). The phytochemicals in the PDB format were converted to standard Simplified Molecule Input Line Entry Specification (SMILES) format and uploaded to the SwissADME prediction tool.^1^ Further, to computationally predict the drug absorption, distribution, metabolism, excretion and toxicity (ADMET) profile of the phytochemicals, the algorithm-based pharmacokinetics server – pkCSM was used.^2^ The server helps in predicting the physiochemical and pharmacological properties of a small molecule. SMILES of the phytochemicals were retrieved from PubChem and uploaded to the pkCSM – pharmacokinetics server. Various *in vivo* absorption parameters like Caco2 permeability, intestinal absorption, P-glycoprotein I and II inhibition were predicted. Drug distribution attributes like penetration of blood–brain barrier (BBB) and central nervous system (CNS), volume of distribution at steady-state (VDss) along with *in vivo* metabolic variables such as inhibition of cytochrome P450 superfamily enzymes were computed. Drug excretion was studied using parameters like total renal clearance and renal OCT2 substrate. Lastly, the toxigenic properties of the phytochemicals were also computationally predicted using the AMES test, hERG I and II inhibition, hepatotoxicity, skin sensitization, and toxicity toward *Tetrahymena pyriformis* and minnows, etc.

### 2.2. Preparation of receptor

The 3D structures of the D13 protein (PDB ID: 6BED, 2.75 Å resolution), A26 protein (PDB ID: 6A9S, Chain A, 1.18 Å resolution), and H3 envelope protein (PDB ID: 5EJ0, Chain A, 1.90 Å resolution) of vaccinia virus were retrieved from the Protein Data Bank (PDB).^3^ All water molecules, heteroatoms, and ligands were removed for the preparation of protein receptor files, followed by the addition of polar hydrogen and Kollman charges. Grid box was generated covering the whole proteins by setting up grid box values as *x* = 27.173, *y* = 3.424, *z* = 33.695 for A26 protein; *x* = 105.514, *y* = 105.444, *z* = 13.868 for D13 protein and *x* = 27.579, *y* = 33.921, *z* = 43.260 for H3 protein. The grid spacing was set to 1 Å.

### 2.3. Preparation of ligands

3D structures of the phytochemicals were downloaded from PubChem in sdf format.^4^ These structures were converted to pdb format using the Open Babel tool. These phytochemicals are known to exhibit antiviral as well as anti-virulent activities (Kumar et al., 2020; Kumar S. et al., 2021; Chadha et al., 2022a). The phytochemicals included in the study are as follows: curcumin (PubChem CID: 969516), E-guggulsterone (PubChem CID: 6439929), licoflavonol (PubChem CID: 5481964), myricetin (PubChem CID: 5281672), oleanolic acid (PubChem CID: 10494), piperidine (PubChem CID: 8082), quercetin (PubChem CID: 5280343), rosmarinic acid (PubChem CID: 5281792), silibinin (PubChem CID: 31553), stigmasterol (PubChem CID: 5280794), ursolic acid (PubChem CID: 64945), withanone (PubChem CID: 21679027). Rifampicin (PubChem CID: 135398735) was used as a positive control for this study.

### 2.4. Molecular docking, analysis, and visualization

AutoDock Vina (Version 1.5.7) was used for the docking studies. The receptors and ligands were prepared in the.pdbqt format. Molecular docking was carried out using instructed command prompt, and the exhaustiveness was set to “24.” Each docking experiment resulted in nine confirmations, and the best binding pose was selected based on the predicted binding energy (in kcal/mol) and molecular interactions. The protein-ligand interactions and PDB structures were visualized and analyzed in PyMOL molecular visualization tool^5^ and LigPlot (Wallace et al., 1995).

### 2.5. Molecular dynamics simulation

Molecular dynamics (MD) simulations were performed for complexes which demonstrated convincing docking score and protein-ligand interactions. Simulations were performed using the GROMACS software (version 2022.4) with GROMOS96 54a7 force field as described earlier (Van Der Spoel et al., 2005; Schmid et al., 2011). Ligand topologies was generated using the PRODRG server (Schüttelkopf and van Aalten, 2004). The docked complex was emplaced in a dodeahedron box with minimum 1 nm distance from the box edge. The system was solvated using SPC water model, followed by neutralization through the addition of 7 sodium ions for the H3 protein, 2 chloride ions for the A26 protein, and 34 sodium ions for the D13 protein. This was followed by energy minimzation to eliminate steric clashes or inappropriate geometry using covergence criteria of maximum force <1,000 kJ/mol/nm. The energy minimized system was subjected to equilibration under NVT and NPT ensembles successively to optimize the temperature and pressure of the system at 300 K and 1 bar, respectively, using the V-rescale thermostat and Parrinello-Rahman pressure coupling. PME was used to compute long-range electrostatic interactions. Following equilibration, unrestrained final production run was performed for a time duration of 100 ns using the leap frog dynamics integrator with a step size of 2 fs. The periodic boundary conditions were considered in three dimensions. The analysis of simulation trajectory was done using modules available in GROMACS. Similar methodology was used for simulation of unliganded protein to serve as control.

### 2.6. MM/PBSA free energy calculation

The MM/PBSA (Molecular Mechanics Poisson Boltzmann Surface Area) technique was utilized for determining the binding energy along with per residue energy contribution. In MM-PBSA, the polar part of the solvation energy ΔGpsolv is calculated by solving the Poisson-Boltzmann equation while ΔGnpsolv is calculated using the linear relation to the solvent accessible surface area. In the present study, g_mmpbsa tool was utilized for the estimation of different components of the binding free energy of complexes (Kumari et al., 2014). The last 10 ns of the trajectory were used for analysis.

### 2.7. Normal mode analysis with iMODS server

Normal mode analysis (NMA) of strongly docked complexes were carried out using iMODS Server^6^ at 300 K constant temperature, 1 atm constant pressure at molecular mechanics level (Jos et al., 2014). iMODS is a computation-based user-friendly server that examines the protein motion within the internal coordinates *via* normal mode analysis. This online server calculates the dihedral coordinates of Cα atoms, structural deformability, and eigenvalues of the docked complex.

### 2.8. Examining the multi-target affinity, cross-reactivity, and inhibition potential of phytochemicals

The LigTMap server was used to map the alternate targets of the virtually screened lead phytochemicals selected for study.^7^ To evaluate drug target specificity, the server provides a ligand similarity score ranging from 0 to 0.9 (0 = lowest, 0.9 = highest) along with the list of target proteins (predicted). The inhibition potential of the lead phytochemcials was also determined in terms of inhibition contant (K*_i_*) from the binding emergy (∆G), using the formula: K*_i_* = (∆G/RT), where R is the universal gas constant (1.985 × 10^–3^ kcal/mol-K) and T is the temperature (298.15 K), as previously described (Ortiz et al., 2019).

## 3. Results

### 3.1. Phytochemical screening and ADME analysis

After undertaking an exhaustive literature survey, we prepared a library of bioactive phytochemicals known to harbor antiviral properties that could possibly be used for inhibiting poxviruses. A total of 12 phytochemicals were shortlisted for the study and further subjected to ADME analysis. All 12 phytochemicals were tested for Lipinski’s rule of five to examine their drug likeliness and physiochemical properties. The rule outlines molecular properties that are important for the pharmacokinetic properties of a drug in the human body, including its absorption, distribution, metabolism, and excretion. These parameters require molecular weight to be <500 Da, the number of hydrogen bond acceptors <10, hydrogen bond donors to be ≤5, and log P, i.e., octanol water coefficient to be <5 (Lipinski, 2004). This rule is important in determining the suitability of the compound to be used as an active drug for oral consumption in humans (Lipinski, 2004). It has been well documented that molecules that fail to follow Lipinski’s rule of five have poor absorption and bioavailability in the body. All the test phytochemicals adhered to Lipinski’s rule of five with no more than 2 violations (at max; Table 1) and displayed ideal drug-like properties, with no toxicity or adverse effects on cellular metabolism (Supplementary Tables S1, S2), thereby being suitable for consumption.

**Table 1:** Lipinski rule of five values for the test phytochemicals.

S. No.	Name of phytochemical	PubChem ID	Molecular weight (< 500 Da)	Lipophilicity (LogP < 5)	#H-bond acceptors (< 10)	#H-bond donors (< 5)	No. of violations
1	Curcumin	969,516	368.38	1.47	6	2	0
2	E-Guggulsterone	6,439,929	312.45	3.86	2	0	0
3	Licoflavone A	5,481,964	322.35	2.2	4	2	0
4	Myricitrin	5,281,672	464.38	−2.32	12	8	2
5	Oleanolic acid	10,494	456.7	5.82	3	2	1
6	Piperine	8,082	285.34	2.39	3	0	0
7	Quercetin	5,280,343	302.24	−0.56	7	5	0
8	Rosmarinic acid	5,281,792	360.31	0.9	8	5	0
9	Silibinin	31,553	482.44	−0.4	10	5	0
10	Stigmasterol	5,280,794	412.69	6.62	1	1	1
11	Ursolic acid	64,945	456.7	5.82	3	2	1
12	Withanone	21,679,027	470.6	2.75	6	2	0

All the compounds were found to obey Lipinski’s rules.

### 3.2. Phytochemicals exhibit high binding affinity toward envelope proteins of poxvirus

**Figure 2:**
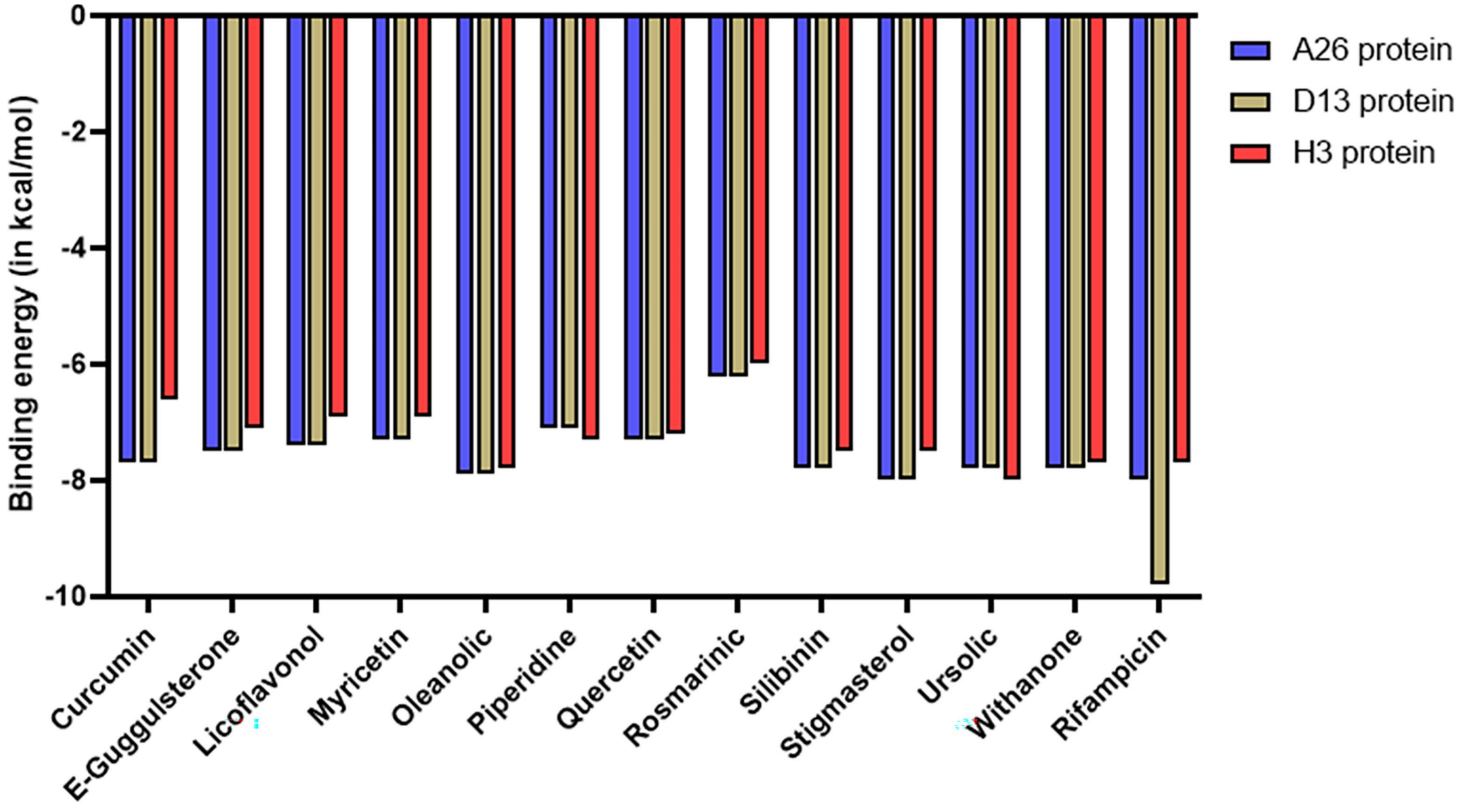
Bar graph depicting the binding energies of different phytochemicals docked against D13, A26, and H3 envelope proteins of MPXV. Rifampicin was used as a control.

Molecular docking was performed to computationally predict the interactions between the phytochemicals and envelope proteins of the vaccinia virus. AutoDock Vina tool was employed to analyze the binding affinities, poses, and confirmation of the ligands with respect to the target receptor. Each docking resulted in 9 different poses for each phytochemical, yielding different binding energies. The pose with the least binding energy (indicating the strongest interaction) was taken into consideration for further analysis. Generally, binding energy lower than the upper threshold of −6.0 kcal/mol is regarded as the cut-off value for determining the robust interaction between a test ligand and protein. All 12 phytochemicals exhibited excellent binding scores with values below −6.0 kcal/mol (Figure 2). Some phytochemicals such as oleanolic acid, ursolic acid, withanone, silibinin, and stigmasterol displayed strong affinities with binding energy even lower than −7.5 kcal/mol against all the 3 protein receptors (Figure 2). The binding energies for most of the phytochemicals were found to be either similar or lower than that of rifampicin, which was used as a control. The results for each envelope protein have been summarized in detail in Supplementary Tables S3–S5 and also discussed in the subsequent sections.

#### 3.2.1. Molecular interactions between D13 protein and phytochemicals

**Figure 3:**
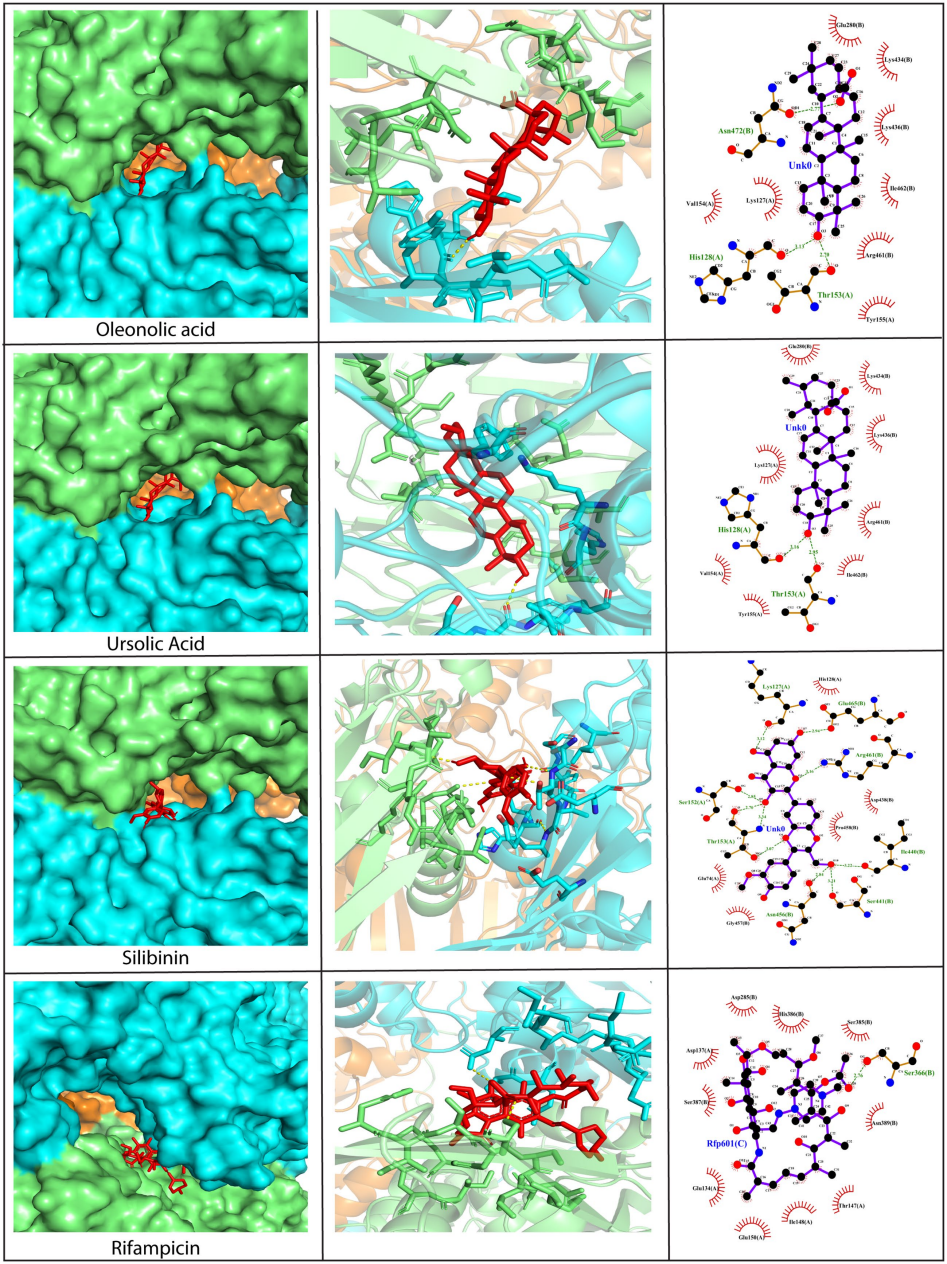
Molecular docking of silibinin, oleanolic acid, and ursolic acid against the D13 protein (PDB ID: 6BED). Left Panel: Visualization of the binding position of the ligand within the protein cavity. Central Panel: Ribbon diagram showing the docked complex. Ligands have been shown in red. Right Panel: 2D diagram representing the interactions between the phytochemicals and D13 protein generated by LigPlot. Dashed lines show hydrogen bonds while the red arcs represent hydrophobic interactions. Rifampicin has been used as a control. Docking was performed *via* AutoDock Vina (Version 1.5.7) and the 3D structures were visualized using PyMol tool.

Since D13 is a viral protein that provides rigidity to the membrane of poxviruses and plays a critical role in viral morphogenesis, it was selected as a drug target for the study. The phytochemicals under investigation demonstrated high binding affinity toward D13 through a plethora of interactions, as compared to rifampicin (Figure 3 and Supplementary Figure S1). The amino acid residues on the D13 protein found to be interacting with the phytochemicals have been enlisted in Supplementary Table S3. Among these, the majority of phytochemicals showed more hydrophobic associations as compared to hydrogen bonds. However, with silibinin and myricetin, the number of hydrogen bonds outnumbered the hydrophobic interactions (Figure 3). Specific residues of the D13 protein, such as Ser152, Thr153, Ser256, and Asn435, significantly facilitated interactions with the phytochemicals. Out of the 12 phytochemicals, oleanolic acid, ursolic acid, and silibinin showed the highest affinity exhibiting binding energies lower than −9.0 kcal/mol with multiple hydrogen bonds and hydrophobic interactions. Briefly, oleanolic acid formed hydrogen bonds with His128, Thr153, and Asn472, and hydrophobic interactions with Lys127, Val154, Tyr155, Glu280, Lys436, Lys434, Arg461, and Ile462. In contrast, ursolic acid showed hydrogen bonding with His128 and Thr153 and hydrophobic interactions with Lys127, Val134, Tyr155, Glu280, Lys434, Lys436, Arg461, and Ileu462. Similarly, silibinin also formed numerous hydrogen bonds with Lys127, Ser152, Thr153, Ile440, Ser441, Asn456, Arg461, and Glu465 along with five hydrophobic interactions with Glu74, His128, Asp438, Gly457, and Pro458. Interestingly, the binding positions of these phytochemicals were similar to that of rifampicin as they occupy the central channel of the D13 trimer. Therefore, it can be speculated that the binding of these phytochemicals at this position may interrupt the assembly of membrane proteins mediated by D13, thereby outlining the potential of phytochemicals as perspective candidates against poxviruses.

#### 3.2.2. Molecular interactions between A26 protein and phytochemicals

**Figure 4:**
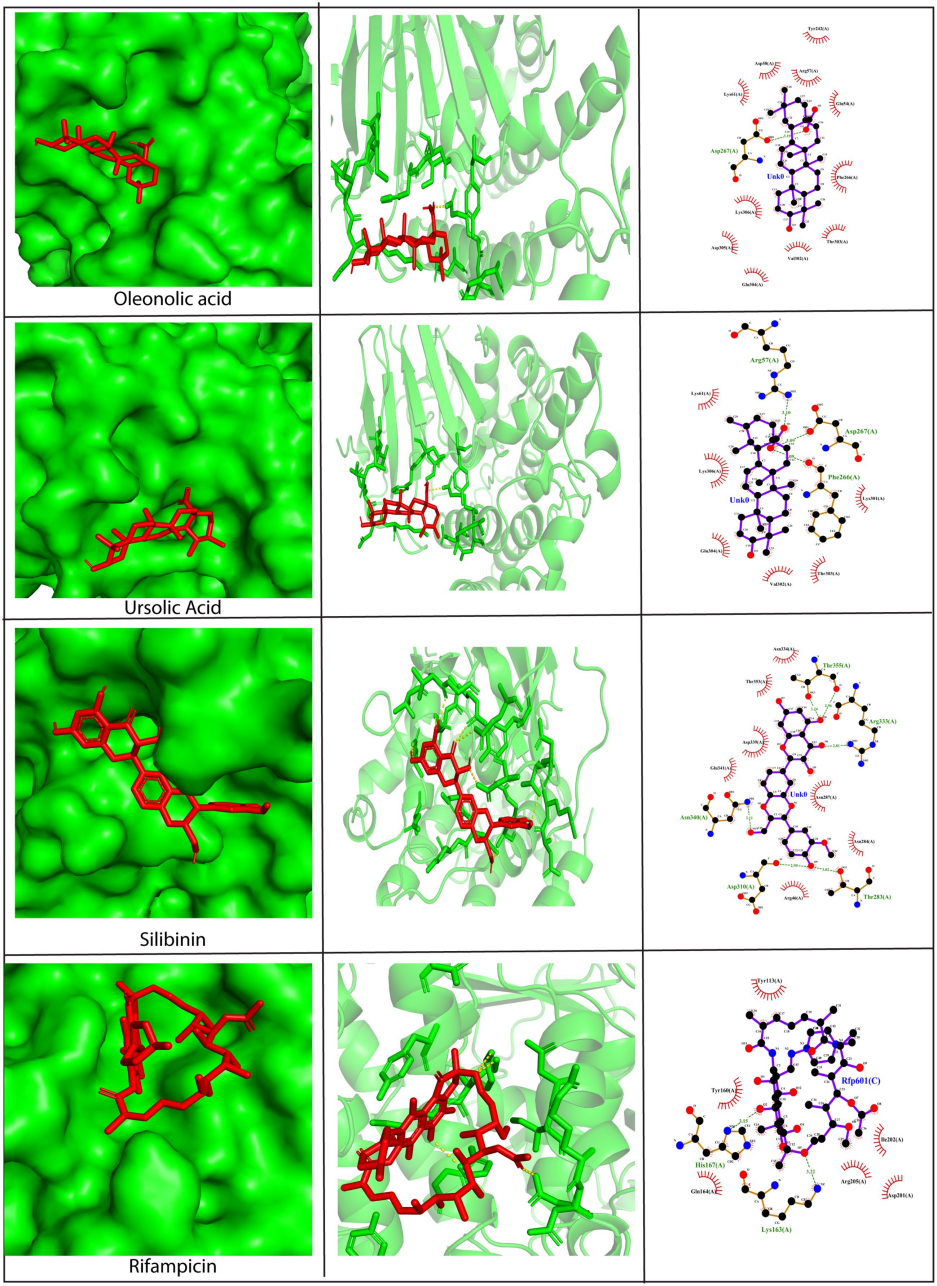
Molecular docking of silibinin, oleanolic acid, and ursolic acid against the A26 protein (PDB ID: 6A9S). Left Panel: Visualization of the binding position of the ligand within the protein cavity. Central Panel: Ribbon diagram showing the docked complex. Ligands are shown in red. Right Panel: 2D diagram representing the interactions between phytochemicals and A26 protein generated by LigPlot. Dashed lines depict hydrogen bonds and the red arcs represent hydrophobic interactions. Rifampicin has been used as a control. Docking was performed *via* AutoDock Vina (Version 1.5.7) and the 3D structures were visualized using PyMol tool.

The A26 protein is a viral envelope protein known to attach to glycosaminoglycans and extracellular matrix laminin on the host cell surface, thereby promoting endocytosis in the vaccinia virus (Carter et al., 2005; Chiu et al., 2007). Hence, its direct inhibition would prevent the entry of viruses into the host cell. To exploit A26 as a drug target, its crystal structure was retrieved from PDB, and blind docking was carried out wherein the docking region covered the entire surface of the protein. Results revealed that all the phytochemicals showed exceptionally low binding scores with values below −7.0 kcal/mol, except rosmarinic acid. Asp267, Thr283, Tyr289, and Asn340 were predicted to be the key amino acid residues involved in mediating interactions with the phytochemicals (Figure 4 and Supplementary Figure S2). The amino acid residues on the A26 protein shown to be interacting with the phytochemicals have been summarized in Supplementary Table S4. The lead compounds that demonstrated efficient binding were stigmasterol (−8.0 kcal/mol), oleanolic acid (−7.9 kcal/mol), ursolic acid (−7.8 kcal/mol), silibinin (−7.8 kcal/mol), and withanone (−7.8 kcal/mol). Stigmasterol was bonded to the A26 protein only *via* hydrophobic bonds, including interactions with the Tyr113, His135, Asp136, Tyr160, and Lys163, Gln164, His167, and Ile 202 residues. On the other hand, ursolic acid formed hydrogen bonds with Arg57, Phe266, and Asp267, and hydrophobic interactions with Lys61, Lys301, Val302, Thr303, Glu304, and Lys306 (Figure 4). Similarly, silibinin formed five hydrogen bonds with Thr283, Asp310, Arg333, Asn340, and Thr355, along with hydrophobic interactions with Arg46, Asn284, Asn287, Asn334, Asp339, Glu341, and Thr353. Withanone also formed five hydrogen bonds with Thr208, Asp310, Lys311, Asp339, and Glu341, and hydrophobic interactions with Asn287, Tyr289, and Asn340 residues. Apart from these phytochemicals, oleanolic acid showed the best binding pose as it formed one hydrogen bond with Asp267 and multiple hydrophobic bonds with Glu54, Arg57, Asp58, Lys61, Tyr242, Phe266, Val301, Thr303, Glu304, Asp305, and Lys306, residues present in the acid-sensing region at the N-terminal region of A26 protein (Figure 4). This region plays a quintessential role in the infectivity of the vaccinia virus (Chang et al., 2019). Based on the docking position of oleanolic acid, it can be hypothesized that it may act as an inhibitor of the A26 protein due to excellent fitting into the N-terminal domain, thereby lowering viral infectivity.

#### 3.2.3. Molecular interactions between H3 protein and phytochemicals

**Figure 5:**
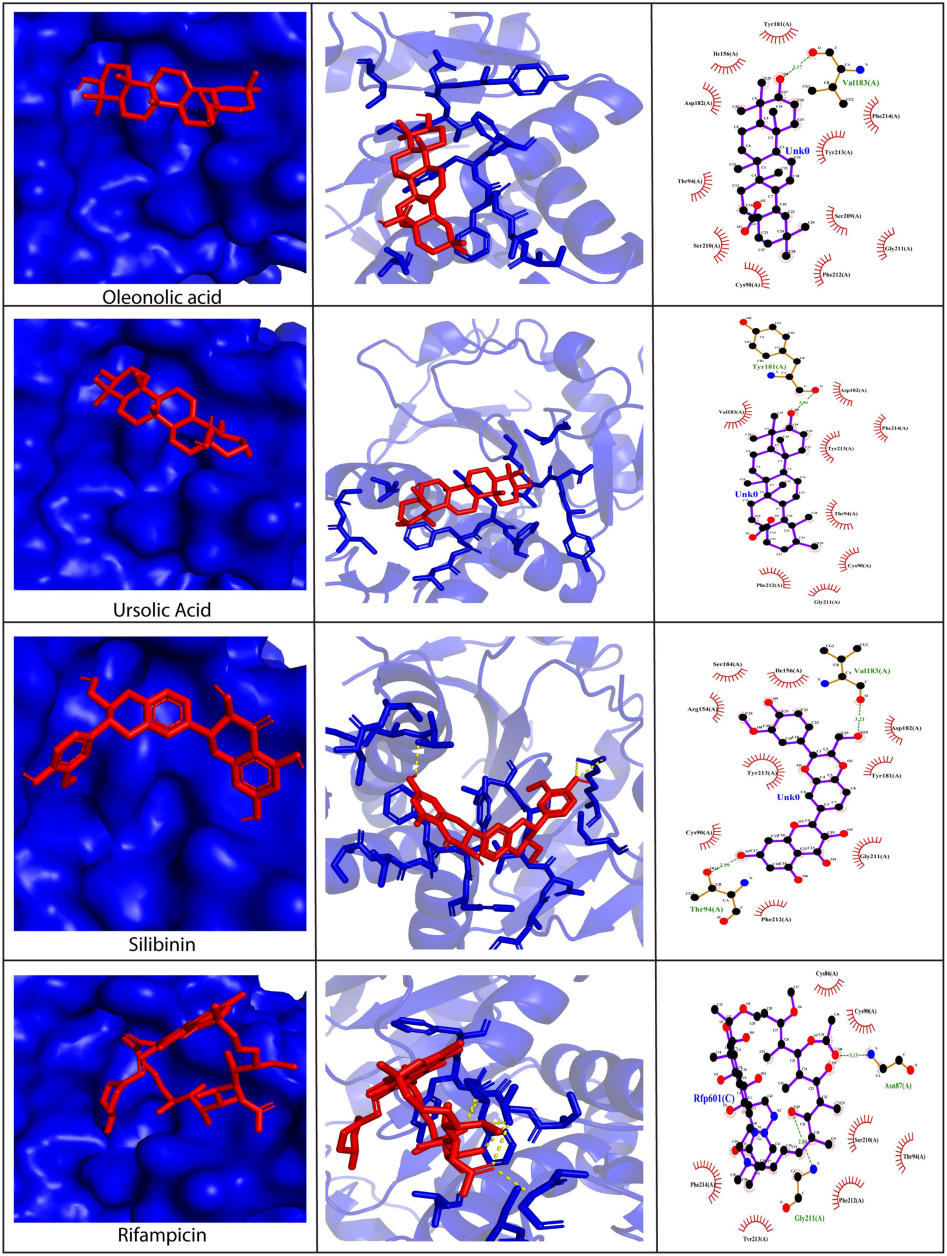
Molecular docking of silibinin, oleanolic acid, and ursolic acid against the H3 protein (PDB ID: 5EJ0). Left Panel: Visualization of the binding position of the ligand within the protein cavity. Central Panel: Ribbon diagram showing the docked complex. Ligands are shown in red. Right Panel: 2D diagram representing the interactions between phytochemicals and H3 protein generated by LigPlot. Dashed lines illustrate hydrogen bonding and the red arcs represent hydrophobic interactions. Rifampicin has been used as a control. Docking was performed *via* AutoDock Vina (Version 1.5.7) and the 3D structures were visualized using PyMol tool.

The H3 protein is another important envelope protein of poxviruses that binds to heparin sulfate on the host cell surface and assists in the virus attachment (Singh et al., 2016). Moreover, it has gained attention among scientists as a potent drug and vaccine target. Hence, we docked bioactive phytochemicals against the H3 viral protein. Our findings revealed that interactions between phytochemicals and the H3 protein were predominantly stabilized by hydrophobic interactions, similar to the case of D13 and A26 proteins (Figure 5 and Supplementary Figure S3). All the predicted interactions between the H3 protein and phytochemicals have been mentioned in Supplementary Table S5. Thr6, Thr91, Thr94, and Val183 were identified as the crucial amino residues facilitating the molecular interactions. Among the 12 phytochemicals, E-guggulsterone, oleanolic acid, piperidine, quercitin, silibinin, stigmasterol, ursolic acid, and withanone displayed excellent binding energies with values below −7.0 kcal/mol. However, the top three compounds with a maximum binding affinity toward the H3 protein were ursolic acid (−8.0 kcal/mol), oleanolic acid (−7.9 kcal/mol), and withanone (−7.2 kcal/mol). Ursolic acid formed one hydrogen bond with Tyr181 and multiple hydrophobic bonds with Cys90, Thr94, Asp182, Val183, Gly211, Phe212, Tyr213, and Phe214 (Figure 5). On similar lines, oleanolic acid formed a lone hydrogen bond with Val183 and numerous hydrophobic bonds with Cys90, Thr94, Ile156, Tyr181, Asp182, Ser209, Ser210, Glu211, Phe212, Tyr213, and Phe214. Withanone also interacted with Thr91 and Thr94 *via* hydrogen bonds and displayed hydrophobic interactions with Cys99, Gly211, Phe212, Tyr213, and Phe214 residues (Figure 5). Taken together, our results reveal that bioactive phytochemicals can bind effectively to the H3 envelope protein of poxviruses.

### 3.3. Molecular dynamics simulations

**Figure 6:**
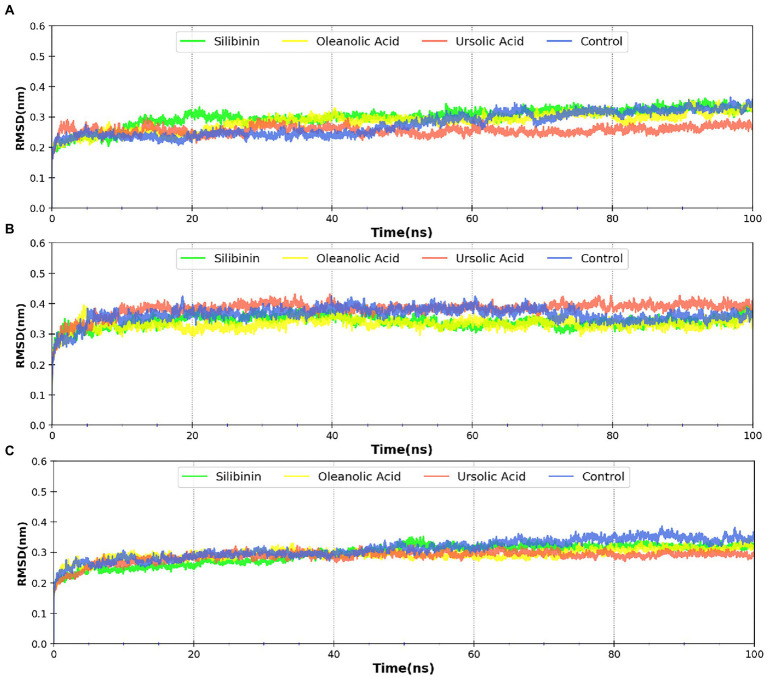
RMSD profile of protein backbone bound to phytomolecules in case of **(A)** A26 protein **(B)** H3 protein and **(C)** D13 protein.

**Figure 7:**
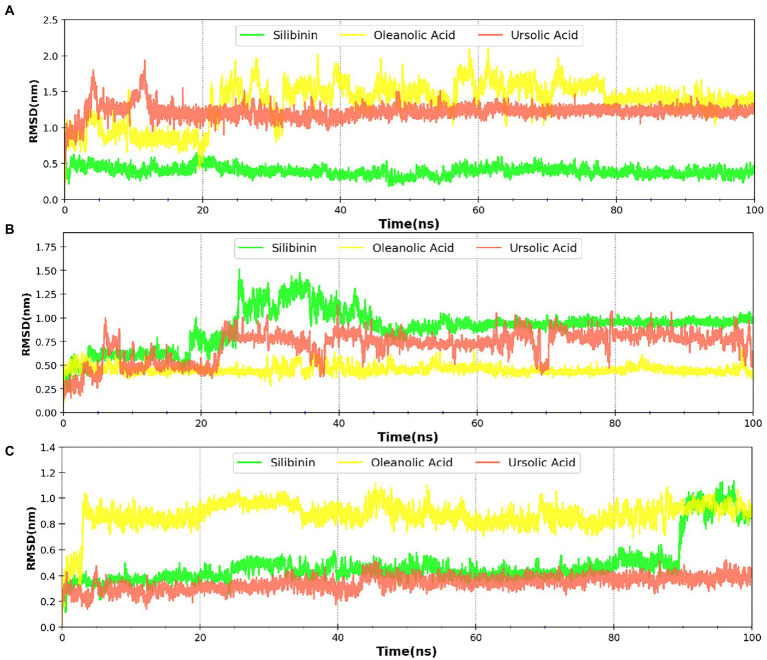
RMSD profile of phytomolecules bound to **(A)** A26 protein **(B)** H3 protein and **(C)** D13 protein.

**Figure 8:**
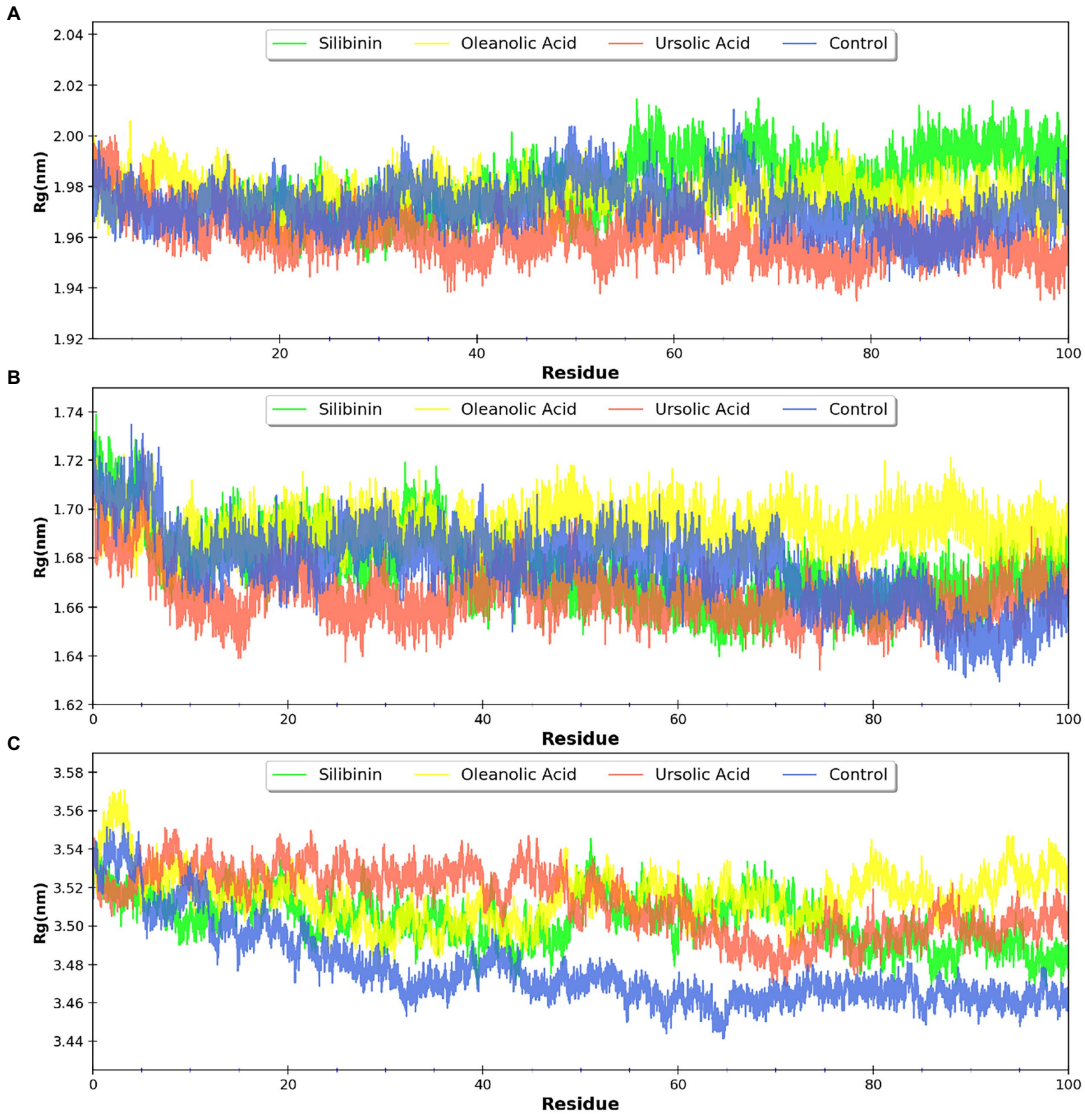
Radius of gyration (Rg) for the proteins bound to phytomolecules **(A)** A26 protein, **(B)** H3 envelop protein, and **(C)** D13 protein.

**Figure 9:**
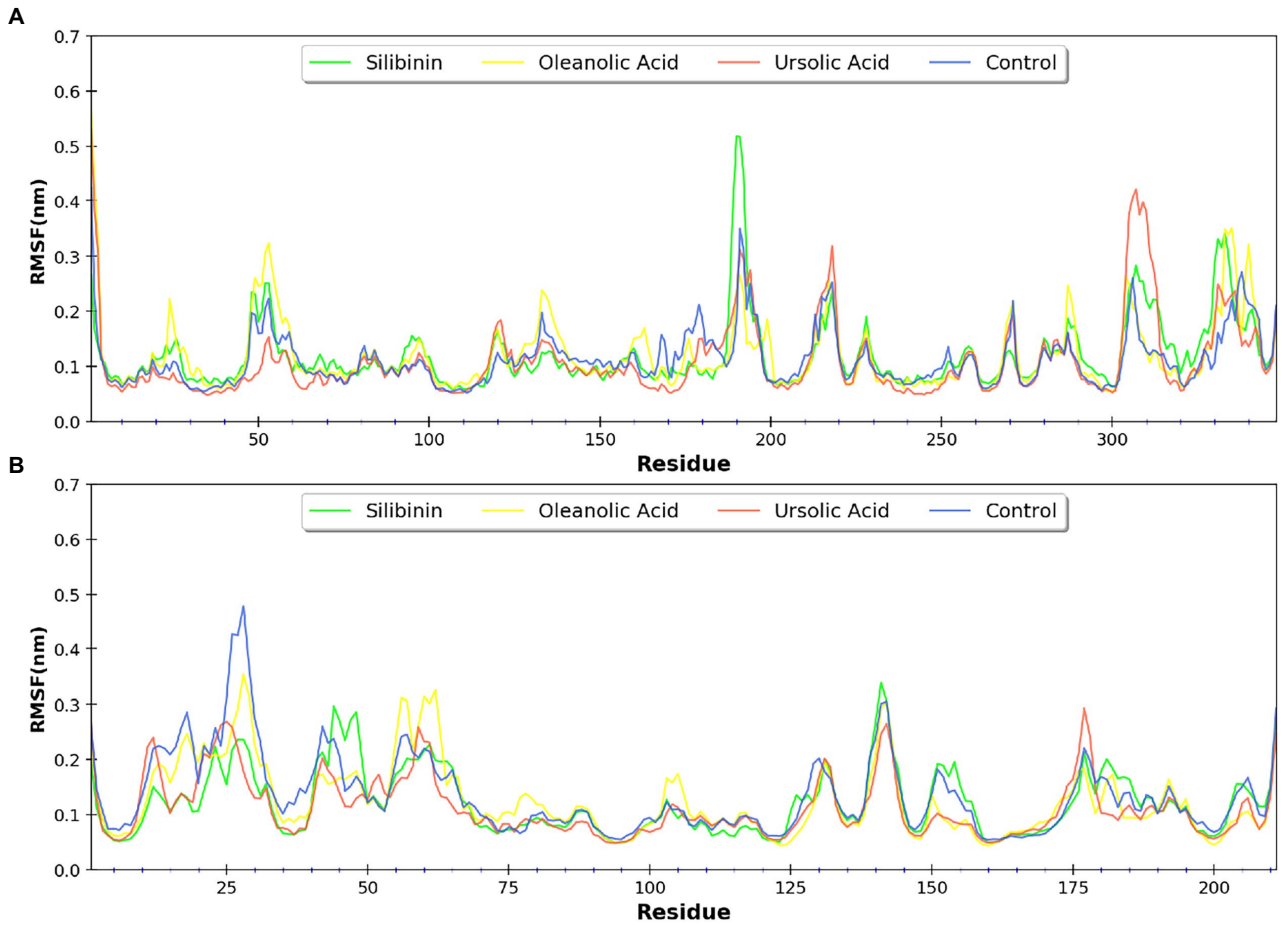
RMSF profile of **(A)** A26 protein and **(B)** H3 protein.

**Figure 10:**
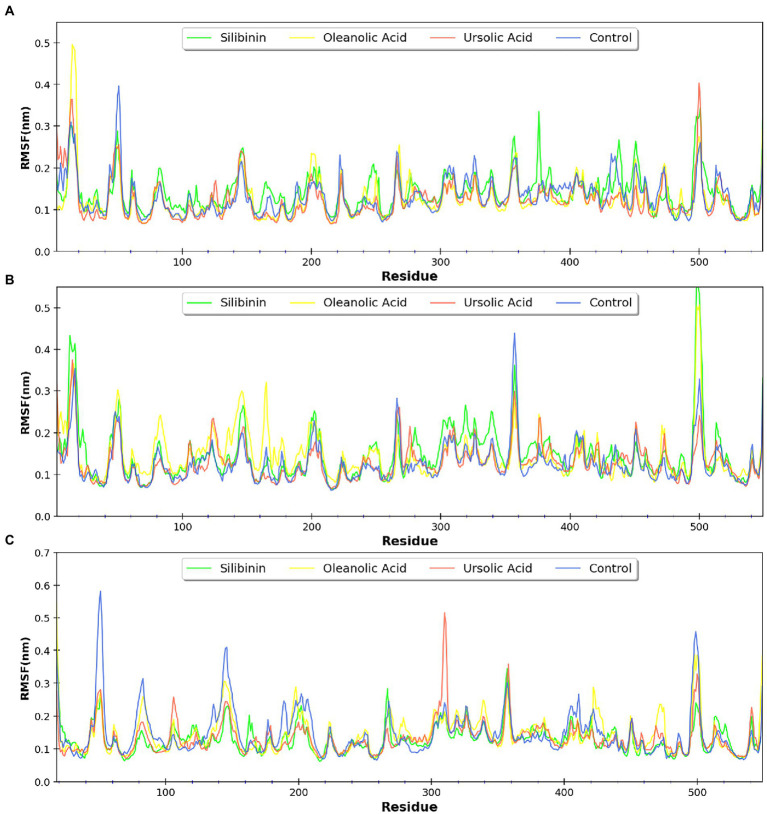
RMSF profile of the three monomers of the D13 protein respresented as **(A–C)**.

MDsimulations were performed in order to assess the dynamic interactions of the selected phytomolecules with the three proteins under aqueous conditions. Structural deviations during the simulations were described in terms of root mean square deviation (RMSD) of atomic coordinates, root mean square fluctuations (RMSF), and radius of gyration of the protein. The backbone RMSD of the proteins in presence of the three phytomolecules was very close to unliganded protein which suggests that binding of the phytomolecules did not impart any major structural change in the envelope proteins (Figure 6). Differences were observed in case of ligand RMSD values although all the protein-ligand complexes were stable throughout the 100 ns simulation (as depicted in Figure 7). The radius of gyration values were similar for all three protein in presence of the phytomolecules, except in the case of D13 protein trimer, where ligand binding lead to an increase in the Rg values (Figure 8). This implies that binding of the phytomolecules reduces the compactness of the trimer. RMSF values in presence of phytomolecules were consistant with the control with variations observed in solvent exposed loop regions (Figures 9, 10). The binding of oleanolic acid to the H3 envelope protein was very stable throughtout 100 ns (Figure 9B). In case of the A26 protein, the binding of oleanolic acid was less stable as it was displaced from it’s docked pose, which was stabilized after 80 ns (Figure 9A). It also showed stable interactions with the hydrophobic cavity between the two monomers of the trimeric D13 (Figure 10). Although stable hydrogen bonds were not observed, the binding was maintained predominantly through hydrophobic interactions. Similar binding modes were observed for ursolic acid with the three receptors as both are pentacyclic triterpenoids. Ursolic acid showed best binding with the trimeric D13 protein. Silibinin being a flavonolignan, has distinct functional groups compared to oleanoic acid and ursolic acid and thus exhibited more diverse interaction capabilities with the three proteins. It showed stable binding with all three proteins by forming hydrogen bonds, π-stacking interactions and other hydrophobic interactions.

### 3.4. MM/PBSA free energy calculation

**Figure 11:**
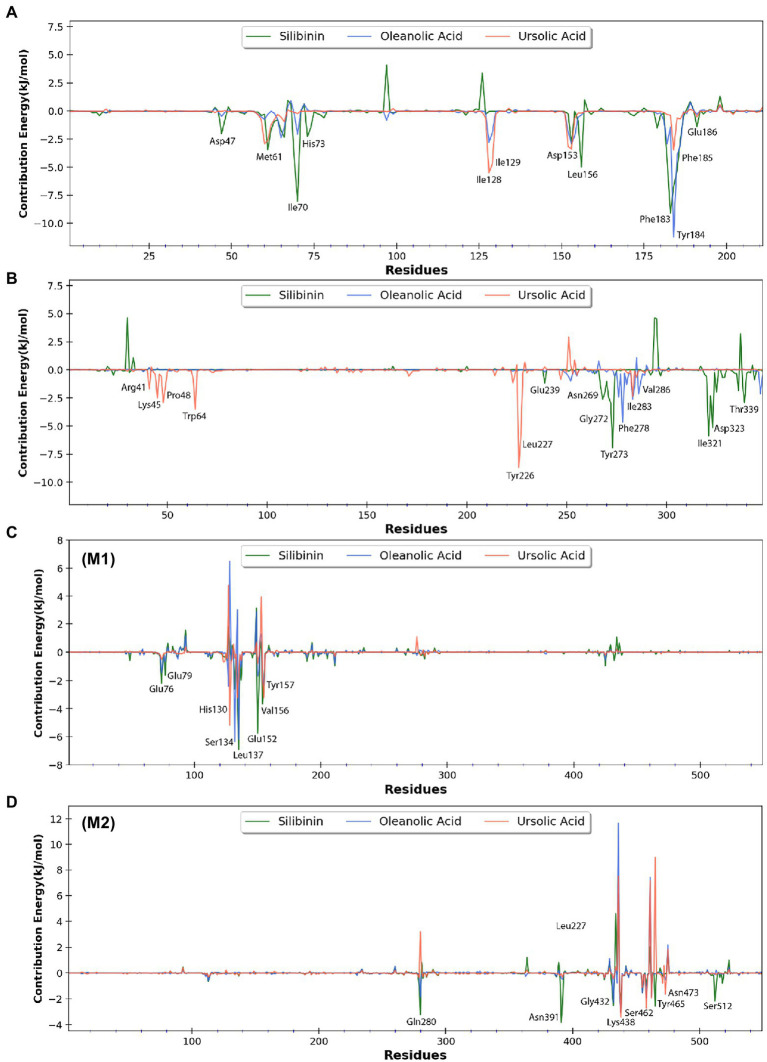
Free energy contribution of **(A)** H3 residues, **(B)** A26 residues, **(C)** D13 residues (monomer-1), and **(D)** D13 residues (monomer-2) interacting with the three selected phytomolecules. Major residues with negative free energy contribution are highlighted.

To quantify the effect of phytomolecules on the activity of the MPX proteins, the free energy was calculated by MM-PBSA analysis (Tables 2–4). The variation of ΔGbind clearly showed that, among all the test molecules, silibinin demonstrates lowest binding energy values. The difference in ΔGbind majorly comes from the ΔEvdw (van der Waals) interaction component and ΔGpsolv (polar part of solvation) free energy components, playing an important role in lower binding free energy. The residue-wise contribution of free energy is depicted in Figure 11, which highlights the interacting residues of the phytomolecules.

**Table 2:** Summary of energy components from MM/PBSA analysis of the lead phytomolecules with the D13 protein.

Name of phytochemical	ΔE_vdw_	+/−	ΔE_EEL_	+/−	ΔG_psolv_	+/−	ΔG_npsolv_	+/−	ΔG_bind_	+/−
Silibinin	−178.798	16.682	−7.729	5.416	65.403	20.664	−18.219	1.603	−139.342	23.111
Oleanolic acid	−169.667	9.077	−6.164	4.028	93.247	11.445	−17.162	1.487	−99.746	12.752
Ursolic acid	−200.753	8.962	−4.753	4.489	114.744	17.493	−17.970	1.078	−108.732	16.563

**Table 3:** Summary of energy components from MM/PBSA analysis of the lead phytomolecules with the A26 protein.

Name of phytochemical	ΔE_vdw_	+/−	ΔE_EEL_	+/−	ΔG_psolv_	+/−	ΔG_npsolv_	+/−	ΔG_bind_	+/−
Silibinin	−207.641	13.822	−4.594	4.522	91.502	13.960	−19.286	1.033	−140.019	16.538
Oleanolic acid	−98.855	18.504	−0.857	2.929	33.213	12.848	−10.255	2.142	−76.754	13.375
Ursolic acid	−124.155	7.587	−1.594	2.501	38.955	6.111	−11.191	1.060	−97.985	8.263

**Table 4:** Summary of energy components from MM/PBSA analysis of the lead phytomolecules with the H3 protein.

Name of phytochemical	ΔE_vdw_	+/−	ΔE_EEL_	+/−	ΔG_psolv_	+/−	ΔG_npsolv_	+/−	ΔG_bind_	+/−
Silibinin	−196.655	14.010	1.587	2.953	48.207	7.487	−18.467	1.250	−165.328	14.763
Oleanolic acid	−129.455	11.025	−2.281	2.629	28.694	6.546	−11.861	1.366	−114.902	10.091
Ursolic acid	−111.655	12.511	0.013	3.350	27.926	10.039	−11.217	1.418	−94.933	11.705

### 3.5. Insights from NMA analysis confirms the stability of phytochemical-protein complexes

Based on the results obtained from molecular docking experiments, we shortlisted three lead phytochemicals *viz*. silibinin, oleanolic acid, and ursolic acid, which demonstrated strong binding affinity toward all the three envelope proteins of poxviruses in common. Subsequently, the stability of the docked phytochemical-protein complexes (best binding pose considered) was examined using MD simulations. The main chain deformability for the complexes has been depicted in the first vertical columns of Supplementary Figure S4 (D13 protein), Supplementary Figure S5 (A26 protein), and Supplementary Figure S6 (H3 protein). Deformability represents the capacity of the given molecule to distort at each residue. Hinges within the plot represent high deformability regions in the protein chain. The second vertical columns of Supplementary Figure S4 (D13 protein), Supplementary Figure S5 (A26 protein), and Supplementary Figure S6 (H3 protein) illustrate the eigenvalues of the docked complexes. Silibinin, oleanolic acid, and ursolic acid demonstrated eigenvalues of 1.825063 × 10^−4^, 1.843076 × 10^−4^, and 1.812478 × 10^−4^ with D13 protein; 6.057804 × 10^−4^, 6.381300 × 10^−4^ and 6.346715 × 10^−4^ with A26 protein; 7.358898 × 10^−4^, 7.377467 × 10^−4^ and 7.618457 × 10^−4^ with H3 protein, respectively. The eigenvalue is a direct measure of the motion stiffness of Cα atoms. The low eigenvalues of the silibinin, oleanolic acid, and ursolic acid with the three structural proteins of poxviruses indicated high stability of the docked complexes. Moreover, the co-variance maps of the complexes (first vertical panel: Supplementary Figures S7–S9), which represents the correlated motion between a pair of residues (red color dots), uncorrelated motion (white color dots), and anti-correlated motion (blue color), also confirmed complex stability and stiffness. This was further substantiated by the elastic network models (second vertical panel: Supplementary Figures S7–S9) that compute the normal modes using a linking matrix to depict the pair of atoms that are connected by springs. The intensity of the gray dots represents the stiffness between the interacting regions. In summary, our findings reveal that the phytochemicals associate strongly with the envelope proteins and demonstrate high stability, yielding satisfactory results from the MD simulations.

### 3.6. Specificity of the phytochemicals to human protein targets

The LigTMap server screened some potential targets for the tested phytochemicals associated with cellular pathway. LigTMap takes into account the chemical configuration of the ligand and the target, pharmacophore properties drug binding properties. For all the lead phytochemicals, the consensus ligand similarity scores were found to be lower than 0.5 (Supplementary Tables S6–S8), thus indicating their target specificity and lower risk of incurring any off-target effects.

## 4. Discussion

With the COVID-19 pandemic slowing down, humankind faces another potential threat due to the sudden emergence of MPXV. The multi-country MPX outbreak may not match the scale of the receeding pandemic but demands immediate global attention and surveillance. With the discontinuation of smallpox vaccine (1980) that provided cross-protection against the MPXV, a large fraction of the world population remains susceptible to MPX (Chadha et al., 2022b). Moreover, the situation has been aggravated by several cluster outbreaks being reported across non-endemic countries and the identification of 50 genomic substitutions within the 2022 MPXV (Chadha et al., 2022b). In view of these events, there is an urgent need to develop therapeutics against the circulating MPXV. In this regard, repurposing bioactive phytochemicals and existing drugs proves to be a promising alternative to combat the wave of MPX infections. Recently, phytochemicals with profound antiviral activity have been successfully repurposed against SARS-CoV-2 (Balkrishna et al., 2021; Lingwan et al., 2021). Hence, the present study was undertaken to virtually screen bioactive phytochemicals targeting the envelope proteins of poxviruses through comprehensive *in silico* analysis. The envelope proteins of vaccinia virus were used as a template for this study since its structural proteins share high sequence similarity with the MPXV (Lam et al., 2022). Although poxviruses possess multiple drug targets, the envelope proteins were chosen specifically in an attempt to prevent virus attachment to the host cell, consequently preventing viral infection at the first instance.

The D13 protein is an essential component of the viral capsid that provides rigidity to the structure of poxviruses, thereby playing an essential role in viral morphogenesis. It is a scaffold protein that forms a honeycomb-like lattice on a viral membrane and aids in forming pleomorphic immature virions in conjunction with A14 and A17 proteins (Van Vliet et al., 2009). Interestingly, the downregulation of the D13 protein results in irregular single bilayer membranes instead of immature virions (Zhang and Moss, 1992). It has been experimentally demonstrated that the D13 protein associates with the viral membrane assembly proteins to form membrane precursors (Maruri-Avidal et al., 2013). Vaccinia virus adopts two modes of entry into the host cell, which include (i) direct fusion with the plasma membrane and (ii) endocytosis. The A26 protein is an envelope protein known to interact with the components of the extracellular matrix of the host cell, thereby promoting acid-dependent endocytosis in vaccinia virus (Bengali et al., 2009). Studies have demonstrated that the loss of the A26 protein blocks the endocytic pathway, thereby triggering the alternate plasma membrane fusion-based route for viral entry into the host cells (Chang et al., 2019). Also, experimental findings by Chang et al. revealed that the loss of the first 75 amino acids from the N-terminal region of the A26 protein in vaccinia virus significantly reduced infectivity as compared to the wild-type strain (Chang et al., 2019). Another important envelope protein of poxviruses is the H3 protein that binds to heparin sulfate residues on the host cell surface, directly facilitating virus attachment (Singh et al., 2016). The crystal structure of the H3 protein shows that it harbors a glycosyltransferase fold and thus binds to UDP glucose (Singh et al., 2016). It is highly conserved and is an immunodominant antigen that is a major target of neutralizing antibodies. Considering the pivotal role of these envelope proteins in the pathogenesis of poxviruses, we selected them as suitable drug targets for this study. Rifampicin was used as a control ligand since it is known to arrest viral morphogenesis before membrane assembly (Grimley et al., 1970). Additionally, rifampicin has been shown to occupy the phenylalanine-rich pocket of the D13 trimer, thereby inhibiting its binding to the A17 protein and affecting the formation of the immature virions (Garriga et al., 2018). However, this binding is reversible, and the assembly of membrane proteins resumes upon withdrawal of the drug. Rifampicin has widely been used as an antibacterial drug as it targets DNA-dependent RNA polymerases. However, its use as an antiviral drug is skeptical in clinical settings because of its low potency and the emergence of resistant viruses.

In the present study, phytochemicals (ligands) were reported to bind effectively to the envelope proteins of MPXV with significantly lower binding energies. The phytochemicals could easily occupy and fit into the active site (pocket) of vaccinia virus A26, D13, and H3 envelope proteins, thus acting as a roadblock for the virus attachment to the host cell. Additionally, the test phytochemicals and control (rifampicin) shared similar molecular interactions with the amino acid residues on the viral proteins. Moreover, with respect to a single protein, certain amino acid residues displayed common interactions with few phytochemicals. Among the 12 compounds screened, 11 displayed binding energies lower than-6.0 kcal/mol against all the three target proteins, except rosmarinic acid, which showed binding energy of −6.0 kcal/mol against the H3 protein. Among the lead phytochemicals, oleanolic acid and ursolic acid belong to the category of pentacyclic triterpenoids. Numerous studies have demonstrated the antiviral, antitumor, and antimicrobial prospects of triterpenoids (Yu et al., 2014; Xiao et al., 2018). Oleanolic acid, an essential component of olive oil, exhibits immunomodulatory effects on the human immune system and has been extensively used in cancer prevention by abrogating various signaling pathways (Ríos, 2010). Numerous studies have also illustrated the application of oleanolic acid and ursolic acid as antiviral agents against different viruses (Khwaza et al., 2018; Shan et al., 2021). Moreover, silibinin is a flavonolignan known to exhibit anti-neoplastic and antioxidative properties. It also inhibits prostate cancer by targeting multiple signaling pathways, inducing apoptosis, and inhibiting metastasis and angiogenesis (Deep and Agarwal, 2010). The existing literature and upcoming investigations collectively increase the foothold for repurposing phytochemicals as alternate therapy against emerging pathogens in clinical settings.

In a recently published study, FDA-approved drugs have also been evaluated for repurposing them against the viral capsid and replication proteins of the vaccinia virus through a comprehensive *in silico* approach (Lam et al., 2022). The role of hydrophobic interactions in stabilizing the protein-ligand complex has been well documented across various investigations (Patil et al., 2010; Ferreira De Freitas and Schapira, 2017). Moreover, hydrogen bondings at the drug-ligand interface are known to play a vital role in stabilizing intermolecular interactions within the complex, thereby promoting complex stiffness (Patil et al., 2010). In this study, a relatively greater number of hydrogen and hydrophobic bonds were formed by the test phytochemicals as compared to the rifampicin. Multiple hydrogen bonds between the phytochemicals and target protein(s) can possibly enhance the stability of the interacting complex. The findings from the molecular docking and counter verification by MD simulation and NMA studies studies collectively provided evidence that bioactive phytochemicals can effectively associate with viral envelope proteins and stimulate conformational changes that may interfere with binding to their cognate ligands on the host cell surface.

Altogether, our data sheds light on the possible molecular interaction(s) between bioactive phytochemicals and the envelope proteins of MPXV, thereby interfering with the functional mechanisms of viral entry into the host cell. The investigation generates preliminary findings that indicate the ability of oleanolic acid, ursolic acid, and silibinin to effectively target the D13, A26, and H3 envelope proteins of MPXV. Nevertheless, these *in silico* findings warrant further experimental validation for their application as alternative medicine against MPX in laboratory settings.

## 5. Conclusion

In summary, of all the phytochemicals tested, oleanolic acid, ursolic acid, and silibinin can be envisioned as the lead compounds for repurposing phytochemicals against poxviruses, including the MPXV. Apart from displaying efficacious binding through a plethora of hydrogen and hydrophobic bonds, these 3 phytochemicals were well-fitted in the pockets of all the three envelope proteins taken into consideration. Thus, these compounds dwell into the criteria of a multi-targeted drug delivery approach which utilizes distinct drug candidates that act synergistically against a particular target through different molecular mechanisms. Owing to their natural existence, these bioactive phytochemicals can be used as alternatives to existing chemical drugs that inflict adverse drug reactions and off-target effects. Hence, this study points toward the possible use of phytochemicals to combat the ongoing multi-country outbreak of MPX. However, this demands further validation through *in vitro* and *in vivo* testing.

## Data availability statement

The original contributions presented in the study are included in the article/Supplementary material, further inquiries can be directed to the corresponding author.

## Author contributions

PG: idea conceptualization, perform molecular docking and MD simulation experiments, data analysis and curation, and manuscript writing. JC: idea conceptualization, experimental design, data curation, manuscript writing and editing, and critical analysis. KH: continuous motivation and formal supervision and analysis. SS: continuous motivation, supervision of the study, and manuscript editing. All authors read and approved the final version of this manuscript.

## Conflict of interest

The authors declare that the research was conducted in the absence of any commercial or financial relationships that could be construed as a potential conflict of interest.

## Publisher’s note

All claims expressed in this article are solely those of the authors and do not necessarily represent those of their affiliated organizations, or those of the publisher, the editors and the reviewers. Any product that may be evaluated in this article, or claim that may be made by its manufacturer, is not guaranteed or endorsed by the publisher.

## Footnotes

^1^ http://www.swissadme.ch

^2^ https://biosig.lab.uq.edu.au/pkcsm/prediction

^3^ https://www.rcsb.org/

^4^ https://pubchem.ncbi.nlm.nih.gov/

^5^ http://www.pymol.org/pymol

^6^ https://imods.iqfr.csic.es/

^7^ https://cbbio.online/LigTMap/

## Supplementary material

SUPPLEMENTARY FIGURE S1 Visualization of molecular docking and interactions between other test phytochemicals docked against the D13 protein (PDB ID: 6BED).

SUPPLEMENTARY FIGURE S2 Visualization of molecular docking and interactions between other test phytochemicals docked against the A26 protein (PDB ID: 6A9S).

SUPPLEMENTARY FIGURE S3 Visualization of molecular docking and interactions between other test phytochemicals docked against the H3 protein (PDB ID:5EJ0).

SUPPLEMENTARY FIGURE S4 Molecular dynamics simulation analysis of silibinin, oleanolic acid, and ursolic acid docked against the D13 protein (PDB ID: 6BED). The left and right panels represent the deformability plot and eigenvalues (plots), respectively.

SUPPLEMENTARY FIGURE S5 Molecular dynamics simulation analysis of silibinin, oleanolic acid, and ursolic acid docked against the A26 protein (PDB ID: 6A9S). The left and right panels represent the deformability plot and eigenvalues (plots), respectively.

SUPPLEMENTARY FIGURE S6 Molecular dynamics simulation analysis of silibinin, oleanolic acid, and ursolic acid docked against the H3 protein (PDB ID: 5EJ0). The left and right panels represent the deformability plot and eigenvalues (plots), respectively.

SUPPLEMENTARY FIGURE S7 Molecular dynamics simulation analysis of silibinin, oleanolic acid, and ursolic acid docked against the D13 protein (PDB ID: 6BED). Left vertical panel shows the covariance matrix. Correlated region has been depicted in red, uncorrelated region in white, and anticorrelated region in blue. Right vertical panel shows elastic network of the docked complexes (dark gray regions indicates more stiffer regions).

SUPPLEMENTARY FIGURE S8 Molecular dynamics simulation analysis of silibinin, oleanolic acid, and ursolic acid docked against the A26 protein (PDB ID: 6A9S). Left vertical panel shows the covariance matrix. Correlated region has been depicted in red, uncorrelated region in white, and anticorrelated region in blue. Right vertical panel shows elastic network of the docked complexes (dark gray regions indicates more stiffer regions).

SUPPLEMENTARY FIGURE S9 Molecular dynamics simulation analysis of silibinin, oleanolic acid, and ursolic acid docked against the H3 protein (PDB ID: 5EJ0). Left vertical panel shows the covariance matrix. Correlated region has been depicted in red, uncorrelated region in white, and anticorrelated region in blue. Right vertical panel shows elastic network of the docked complexes (dark gray regions indicates more stiffer regions).

## References

[ref1] Balkrishna A. Pokhrel S. Singh H. Joshi M. Mulay V. P. Haldar S.. (2021). Withanone from *Withania somnifera* attenuates SARS-CoV-2 RBD and host ACE2 interactions to rescue spike protein induced pathologies in humanized zebrafish model. Drug Des. Devel. Ther. 15, 1111–1133. doi: 10.2147/DDDT.S292805, PMID: 33737804 PMC7961299

[ref2] Benet L. Z. Hosey C. M. Ursu O. Oprea T. I. (2016). BDDCS, the rule of 5 and drugability. Adv. Drug Deliv. Rev. 101, 89–98. doi: 10.1016/J.ADDR.2016.05.007, PMID: 27182629 PMC4910824

[ref3] Bengali Z. Townsley A. C. Moss B. (2009). Vaccinia virus strain differences in cell attachment and entry. Virology 389, 132–140. doi: 10.1016/J.VIROL.2009.04.012, PMID: 19428041 PMC2700833

[ref4] Bunge E. M. Hoet B. Chen L. Lienert F. Weidenthaler H. Baer L. R.. (2022). The changing epidemiology of human monkeypox—a potential threat? A systematic review. PLoS Negl. Trop. Dis. 16:e0010141. doi: 10.1371/JOURNAL.PNTD.0010141, PMID: 35148313 PMC8870502

[ref5] Carter G. C. Law M. Hollinshead M. Smith G. L. (2005). Entry of the vaccinia virus intracellular mature virion and its interactions with glycosaminoglycans. J. Gen. Virol. 86, 1279–1290. doi: 10.1099/VIR.0.80831-0, PMID: 15831938

[ref6] Chadha J. Gupta M. Nagpal N. Sharma M. Adarsh T. Joshi V.. (2021). Antibacterial potential of indigenous plant extracts against multidrug-resistant bacterial strains isolated from New Delhi region. GSC Biol. Pharm. Sci. 14, 185–196. doi: 10.30574/GSCBPS.2021.14.2.0053

[ref7] Chadha J. Harjai K. Chhibber S. (2022a). Repurposing phytochemicals as anti-virulent agents to attenuate quorum sensing-regulated virulence factors and biofilm formation in *Pseudomonas aeruginosa*. Microb. Biotechnol. 15, 1695–1718. doi: 10.1111/1751-7915.13981, PMID: 34843159 PMC9151347

[ref8] Chadha J. Khullar L. Gulati P. Chhibber S. Harjai K. (2022b). Insights into the monkeypox virus: making of another pandemic within the pandemic? Environ. Microbiol. 24, 4547–4560. doi: 10.1111/1462-2920.16174, PMID: 35974453

[ref9] Chadha J. Khullar L. Mittal N. (2022c). Facing the wrath of enigmatic mutations: a review on the emergence of severe acute respiratory syndrome coronavirus 2 variants amid coronavirus disease-19 pandemic. Environ. Microbiol. 24, 2615–2629. doi: 10.1111/1462-2920.15687, PMID: 34320263 PMC8441773

[ref10] Chang H. W. Yang C. H. Luo Y. C. Su B. G. Cheng H. Y. Tung S. Y.. (2019). Vaccinia viral A26 protein is a fusion suppressor of mature virus and triggers membrane fusion through conformational change at low pH. PLoS Pathog. 15:e1007826. doi: 10.1371/JOURNAL.PPAT.1007826, PMID: 31220181 PMC6605681

[ref11] Chiu W.-L. Lin C.-L. Yang M.-H. Tzou D.-L. M. Chang W. (2007). Vaccinia virus 4c (A26L) protein on intracellular mature virus binds to the extracellular cellular matrix laminin. J. Virol. 81, 2149–2157. doi: 10.1128/JVI.02302-06, PMID: 17166913 PMC1865921

[ref12] Choudhury A. Das N. C. Patra R. Bhattacharya M. Ghosh P. Patra B. C.. (2021). Exploring the binding efficacy of ivermectin against the key proteins of SARS-CoV-2 pathogenesis: an approach. Future Virol. 16, 277–291. doi: 10.2217/FVL-2020-0342

[ref13] Daina A. Michielin O. Zoete V. (2017). SwissADME: a free web tool to evaluate pharmacokinetics, drug-likeness and medicinal chemistry friendliness of small molecules. Sci. Rep. 7, 1–13. doi: 10.1038/srep42717, PMID: 28256516 PMC5335600

[ref14] Deep G. Agarwal R. (2010). Antimetastatic efficacy of silibinin: molecular mechanisms and therapeutic potential against cancer. Cancer Metastasis Rev. 2010 293, 447–463. doi: 10.1007/S10555-010-9237-0 PMC3928361 20714788

[ref15] Ferreira De Freitas R. Schapira M. (2017). A systematic analysis of atomic protein–ligand interactions in the PDB. Medchemcomm 8, 1970–1981. doi: 10.1039/C7MD00381A, PMID: 29308120 PMC5708362

[ref16] Garriga D. Headey S. Accurso C. Gunzburg M. Scanlon M. Coulibaly F. (2018). Structural basis for the inhibition of poxvirus assembly by the antibiotic rifampicin. Proc. Natl. Acad. Sci. U. S. A. 115, 8424–8429. doi: 10.1073/PNAS.1810398115, PMID: 30068608 PMC6099865

[ref17] Grimley P. M. Rosenblum E. N. Mims S. J. Moss B. (1970). Interruption by rifampin of an early stage in vaccinia virus morphogenesis: accumulation of membranes which are precursors of virus envelopes. J. Virol. 6, 519–533. doi: 10.1128/JVI.6.4.519-533.1970, PMID: 5497899 PMC376151

[ref18] Gulati P. Yadav A. Chadha J. Singh S. (2021). Virtual screening of phytochemicals targeting the Main protease and spike protein of SARS-CoV-2: an in silico approach. J. Biomed. Res. Environ. Sci. 2, 1121–1131. doi: 10.37871/JBRES1357

[ref19] Isidro J. Borges V. Pinto M. Sobral D. Santos J. D. Nunes A.. (2022). Phylogenomic characterization and signs of microevolution in the 2022 multi-country outbreak of monkeypox virus. Nat. Med. 28, 1569–1572. doi: 10.1038/s41591-022-01907-y, PMID: 35750157 PMC9388373

[ref20] Jos J. Ramon J. Ramon R. Opez-Blanco L. Aliaga J. I. Quintana-Ortí E. S.. (2014). iMODS: internal coordinates normal mode analysis server. Nucleic Acids Res. 42, W271–W276. doi: 10.1093/NAR/GKU339, PMID: 24771341 PMC4086069

[ref21] Khwaza V. Oyedeji O. O. Aderibigbe B. A. Ciminiello P. Mangoni A. Menna M.. (2018). Antiviral activities of oleanolic acid and its analogues. Molecules 23:2300. doi: 10.3390/MOLECULES23092300, PMID: 30205592 PMC6225463

[ref22] Kugelman J. R. Johnston S. C. Mulembakani P. M. Kisalu N. Lee M. S. Koroleva G.. (2014). Genomic variability of monkeypox virus among humans, Democratic Republic of the Congo. Emerg. Infect. Dis. 20, 232–239. doi: 10.3201/EID2002.130118, PMID: 24457084 PMC3901482

[ref23] Kumar V. Dhanjal J. K. Bhargava P. Kaul A. Wang J. Zhang H.. (2020). Withanone and Withaferin-a are predicted to interact with transmembrane protease serine 2 (TMPRSS2) and block entry of SARS-CoV-2 into cells. J. Biomol. Struct. Dyn. 40, 1–13. doi: 10.1080/07391102.2020.1775704, PMID: 32469279 PMC7309304

[ref24] Kumar S. Garg C. Kaushik S. Buttar H. Garg M. (2021). Demystifying therapeutic potential of medicinal plants against chikungunya virus. Indian J. Pharmacol. 53, 403–411. doi: 10.4103/IJP.IJP_81_20, PMID: 34854411 PMC8641736

[ref25] Kumar A. Mishra D. C. Angadi U. B. Yadav R. Rai A. Kumar D. (2021). Inhibition potencies of phytochemicals derived from sesame against SARS-CoV-2 main protease: a molecular docking and simulation study. Front. Chem. 9:773. doi: 10.3389/FCHEM.2021.744376, PMID: 34692642 PMC8531729

[ref26] Kumari R. Kumar R Open Source Drug Discovery Consortium Lynn A. (2014). g_mmpbsa--a GROMACS tool for high-throughput MM-PBSA calculations. J. Chem. Inf. Model. 54:1951–1962. doi: 10.1021/ci500020m 24850022

[ref27] Kwofie S. K. Broni E. Teye J. Quansah E. Issah I. Wilson M. D.. (2019). Pharmacoinformatics-based identification of potential bioactive compounds against Ebola virus protein VP24. Comput. Biol. Med. 113:103414. doi: 10.1016/J.COMPBIOMED.2019.103414, PMID: 31536833

[ref28] Lam H. Y. I. Guan J. S. Mu Y. (2022). In silico repurposed drugs against monkeypox virus. Molecules 27, 1–20. doi: 10.3390/MOLECULES27165277/S1, PMID: 36014515 PMC9415168

[ref29] Ligon B. L. (2004). Monkeypox: a review of the history and emergence in the Western hemisphere. Semin. Pediatr. Infect. Dis. 15, 280–287. doi: 10.1053/J.SPID.2004.09.001, PMID: 15494953 PMC7129998

[ref30] Lingwan M. Shagun S. Pahwa F. Kumar A. Verma D. K. Pant Y.. (2021). Phytochemical rich Himalayan Rhododendron arboreum petals inhibit SARS-CoV-2 infection in vitro. J. Biomol. Struct. Dyn., 1–11. doi: 10.1080/07391102.2021.2021287, PMID: 34961411

[ref31] Lipinski C. A. (2004). Lead-and drug-like compounds: the rule-of-five revolution. Drug Discov. Today Technol. 1, 337–341. doi: 10.1016/J.DDTEC.2004.11.007, PMID: 24981612

[ref32] Marennikova S. S. Seluhina E. M. Malceva N. N. Cimiskjan K. L. Macevic G. R. (1972). Isolation and properties of the causal agent of a new variola-like disease (monkeypox) in man. Bull. World Health Organ. 46, 599–611. PMID: 4340219 PMC2480798

[ref33] Maruri-Avidal L. Weisberg A. S. Moss B. (2013). Direct formation of vaccinia virus membranes from the endoplasmic reticulum in the absence of the newly characterized L2-interacting protein A30.5. J. Virol. 87, 12313–12326. doi: 10.1128/JVI.02137-13, PMID: 24027302 PMC3807895

[ref34] Mourya D. Yadav P. Ullas P. Bhardwaj S. Sahay R. Chadha M.. (2019). Emerging/re-emerging viral diseases & new viruses on the Indian horizon. Indian J. Med. Res. 149, 447–467. doi: 10.4103/IJMR.IJMR_1239_18, PMID: 31411169 PMC6676836

[ref35] Ortiz C. L. D. Completo G. C. Nacario R. C. Nellas R. B. (2019). Potential inhibitors of Galactofuranosyltransferase 2 (GlfT2): molecular docking, 3D-QSAR, and in silico ADMETox studies. Sci. Rep. 9, 17096–17028. doi: 10.1038/s41598-019-52764-8, PMID: 31745103 PMC6863818

[ref36] Pathania S. Randhawa V. Kumar M. (2019). Identifying potential entry inhibitors for emerging Nipah virus by molecular docking and chemical-protein interaction network. J. Biomol. Struct. Dyn. 38, 5108–5125. doi: 10.1080/07391102.2019.1696705, PMID: 31771426

[ref37] Patil R. Das S. Stanley A. Yadav L. Sudhakar A. Varma A. K. (2010). Optimized hydrophobic interactions and hydrogen bonding at the target-ligand Interface leads the pathways of drug-designing. PLoS One 5:e12029. doi: 10.1371/JOURNAL.PONE.0012029, PMID: 20808434 PMC2922327

[ref38] Reynolds M. G. Carroll D. S. Karem K. L. (2012). Factors affecting the likelihood of monkeypox’s emergence and spread in the post-smallpox era. Curr. Opin. Virol. 2, 335–343. doi: 10.1016/J.COVIRO.2012.02.004, PMID: 22709519 PMC9533834

[ref39] Ríos J. L. (2010). Effects of triterpenes on the immune system. J. Ethnopharmacol. 128, 1–14. doi: 10.1016/J.JEP.2009.12.045 20079412

[ref40] Schmid N. Eichenberger A. P. Choutko A. Riniker S. Winger M. Mark A. E.. (2011). Definition and testing of the GROMOS force-field versions 54A7 and 54B7. Eur. Biophys. J. 40, 843–856. doi: 10.1007/s00249-011-0700-9, PMID: 21533652

[ref41] Schüttelkopf A. W. van Aalten D. M. (2004). PRODRG: a tool for high-throughput crystallography of protein-ligand complexes. Acta Crystallogr. D Biol. Crystallogr. 60, 1355–1363. doi: 10.1107/S0907444904011679, PMID: 15272157

[ref42] Shan T. Ye J. Jia J. Wang Z. Jiang Y. Wang Y.. (2021). Viral UL8 is involved in the antiviral activity of oleanolic acid against HSV-1 infection. Front. Microbiol. 12:2046. doi: 10.3389/FMICB.2021.689607, PMID: 34354687 PMC8329587

[ref43] Singh K. Gittis A. G. Gitti R. K. Ostazeski S. A. Su H.-P. Garboczi D. N. (2016). The vaccinia virus H3 envelope protein, a major target of neutralizing antibodies, exhibits a glycosyltransferase fold and binds UDP-glucose. J. Virol. 90, 5020–5030. doi: 10.1128/JVI.02933-15, PMID: 26937025 PMC4859701

[ref44] Sklenovská N. Van Ranst M. (2018). Emergence of Monkeypox as the most important Orthopoxvirus infection in humans. Front. Public Heal. 6:241. doi: 10.3389/FPUBH.2018.0024, PMID: 30234087 PMC6131633

[ref45] Titanji B. K. Tegomoh B. Nematollahi S. Konomos M. Kulkarni P. A. (2022). Monkeypox: a contemporary review for healthcare professionals. Open Forum Infect. Dis. 9:ofac310. doi: 10.1093/OFID/OFAC310, PMID: 35891689 PMC9307103

[ref46] Van Der Spoel D. Lindahl E. Hess B. Groenhof G. Mark A. E. Berendsen H. J. (2005). GROMACS: fast, flexible, and free. J. Comput. Chem. 26, 1701–1718. doi: 10.1002/jcc.20291, PMID: 16211538

[ref47] Van Vliet K. Mohamed M. R. Zhang L. Villa N. Y. Werden S. J. Liu J.. (2009). Poxvirus proteomics and virus-host protein interactions. Microbiol. Mol. Biol. Rev. 73, 730–749. doi: 10.1128/MMBR.00026-09, PMID: 19946139 PMC2786582

[ref48] Wallace A. C. Laskowski R. A. Thornton J. M. (1995). LIGPLOT: a program to generate schematic diagrams of protein-ligand interactions. Protein Eng. Des. Sel. 8, 127–134. doi: 10.1093/PROTEIN/8.2.127 7630882

[ref90] WHO (2022). Monkeypox. World Health Organization. https://www.who.int/news-room/fact-sheets/detail/monkeypox [Accessed 10th October 2022].

[ref49] Xiao S. Tian Z. Wang Y. Si L. Zhang L. Zhou D. (2018). Recent progress in the antiviral activity and mechanism study of pentacyclic triterpenoids and their derivatives. Med. Res. Rev. 38, 951–976. doi: 10.1002/MED.21484, PMID: 29350407 PMC7168445

[ref50] Yu M. Si L. Wang Y. Wu Y. Yu F. Jiao P.. (2014). Discovery of pentacyclic triterpenoids as potential entry inhibitors of influenza viruses. J. Med. Chem. 57, 10058–10071. doi: 10.1021/JM5014067, PMID: 25383779

[ref51] Zhang Y. Moss B. (1992). Immature viral envelope formation is interrupted at the same stage by lac operator-mediated repression of the vaccinia virus D13L gene and by the drug rifampicin. Virology 187, 643–653. doi: 10.1016/0042-6822(92)90467-4, PMID: 1546459

