# Supplementary material for: Targeting envelope proteins of poxviruses to repurpose phytochemicals against monkeypox: An in silico investigation
Source: Front Microbiol. 2023 Jan 5;13:1073419. doi: 10.3389/fmicb.2022.1073419 (PMC9849581; doi:10.3389/fmicb.2022.1073419)
Supplement: SUPPLEMENTARY FIGURE S1 — Visualization of molecular docking and interactions between other test phytochemicals docked against the D13 protein (PDB ID: 6BED). [file Data_Sheet_1.PDF]

Docked complex	Interactions
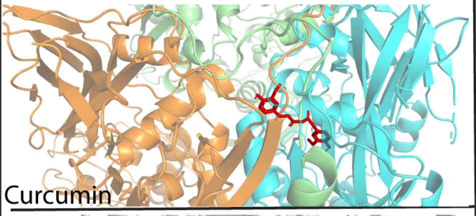 Curcumin	Hydrophobic Bonds: Phe481,Leu467,Thr478,Asn480,Thr468,Ser256,Ser254,Tyr258<br>Hydrogen Bonds: Asp166,Asn464,Thr474,Glu114,Asn117
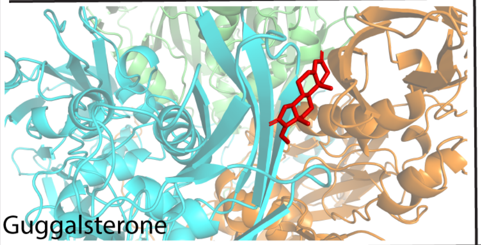 Guggalsterone	Hydrophobic Bonds: Val315,Val330,Pro316,Ile332,Ala375,Asn334,Asp333,Asp374, Val335,Ser350<br>Hydrogen Bonds: Val341
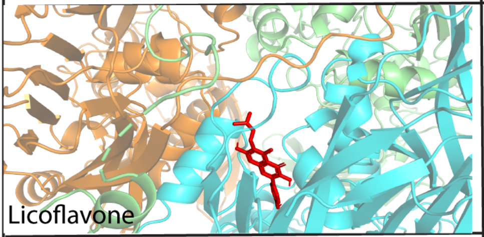 Licoflavone	Hydrophobic Bonds: Asn480,Ser254,Val528,Ser256,Tyr258<br>Hydrogen Bonds: Thr478,Glu230,Lys484,Asn530,Glu114
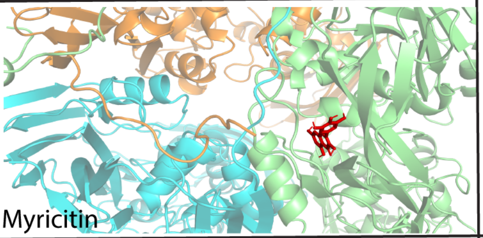 Myricitin	Hydrophobic Bonds: Gly473,Asn464,Ile120,Asn121,Asn117<br>Hydrogen Bonds: Thr474,Thr476,Ser256,Glu230,Lys484,Asn530,Asn118,Ser470
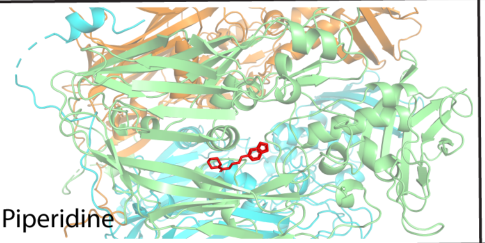 Piperidine	Hydrophobic Bonds: Asn435,Lys429,Asn472,Ile120,Gly473,Asn117,Asn484,Thr468,Ser470<br>Hydrogen Bonds: Tyr117,Thr474
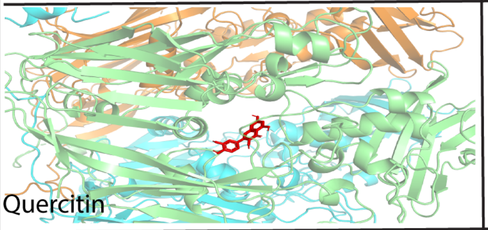 Quercitin	Hydrophobic Bonds: Pro88,Tyr89,Tyr116,Phe433,Ile428,Phe427,Asn435<br>Hydrogen Bonds: Asn117,Gln278,Lys429
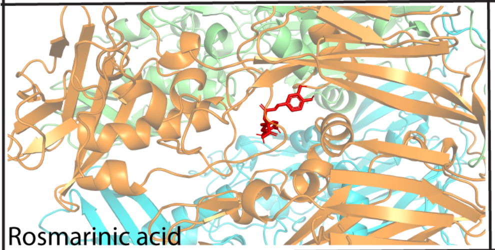 Rosmarinic acid	Hydrophobic Bonds: Thr474,Asn117,Ile428,Gly473,Ile120,Asn435,Phe427,Lys434,Phe433, Lys429,Lys93,Asn472,Ile120<br>Hydrogen Bonds: Tyr89,Tyr116,Asp425,Asn472
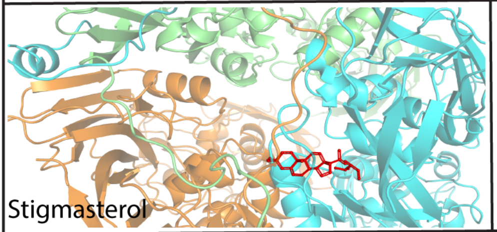 Stigmasterol	Hydrophobic Bonds: Asp166,Asn480,Phe481,Lys484,Asn464,Thr478,Thr468,Thr474 Asn472,Asn117,Ser256,Asn118
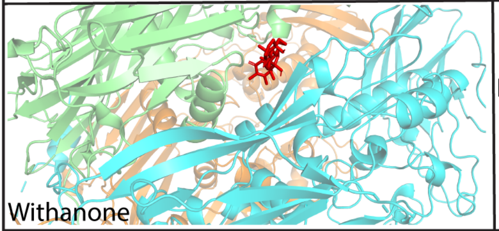 Withanone	Hydrophobic Bonds: Lys127,Glu465,Gly457,Asn456,Pro458<br>Hydrogen Bonds: Thr153,Tyr155,Ser152,Arg461

Docked complex	Interactions
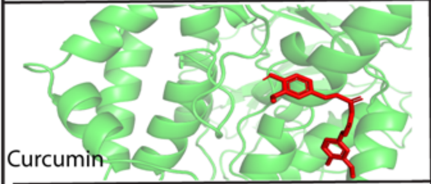 Curcumin	Hydrophobic Bonds: Lys163,His167,Tyr160,Glu156,Asp136<br>Hydrogen Bonds: Thr138,Asn134,Pro139,Arg157
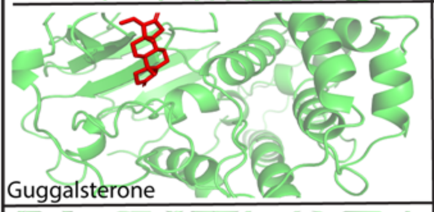 Guggalsterone	Hydrophobic Bonds: Thr283,Lys311,Asn340,Asp310,Asn284,Asn285,Ile337<br>Hydrogen Bonds: Tyr289,Arg46
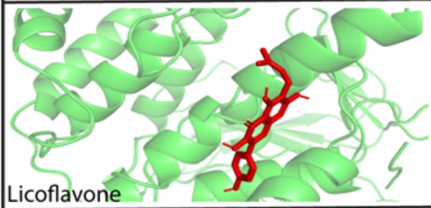 Licoflavone	Hydrophobic Bonds: Thr160,Arg157,Arg205,Ile202,His167<br>Hydrogen Bonds: Glu156,Lys163,Asp201
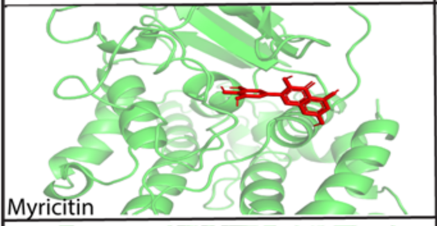 Myricitin	Hydrophobic Bonds: Ile337,Asn340,Thr283,Lys311<br>Hydrogen Bonds: Asp339,Tyr289,Arg46,Asp310
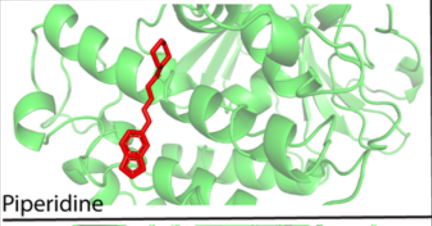 Piperidine	Hydrophobic Bonds: His135,Tyr113,Asp136,Tyr160,Gln164,Ile202,Lys163,Asp201,Thr303<br>Hydrogen Bonds:
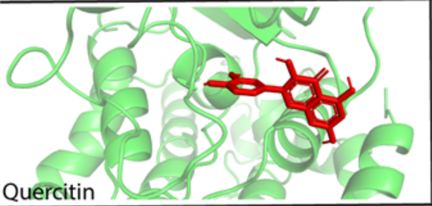 Quercitin	Hydrophobic Bonds: Asn340,Ile337,Thr283,Lys311<br>Hydrogen Bonds: Tyr289,Asp339,Ile338,Arg46,Asp310
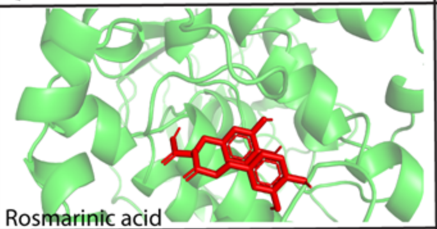 Rosmarinic acid	Hydrophobic Bonds: Thr109,Asp136,Leu110,Asn111,Gly137,Tyr124,Phe107<br>Hydrogen Bonds: Lys108,His135,Lys133
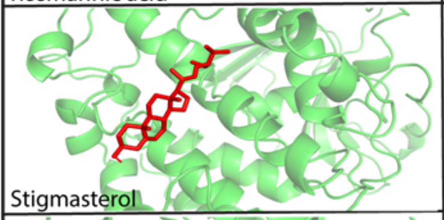 Stigmasterol	Hydrophobic Bonds: Ile202,His167,Lys163,Tyr160,Gln164,His135,Tyr113,Asp136<br>Hydrogen Bonds:
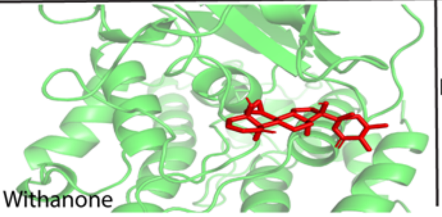 Withanone	Hydrophobic Bonds: Glu341,Thr283,Asp339,Asp310,Lys311<br>Hydrogen Bonds: Asn287,Tyr289,Asn340

Docked complex	Interactions
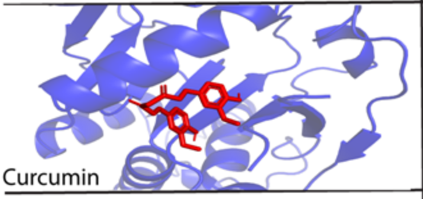 Curcumin	Hydrophobic Bonds: Tyr62,Arg137,Lys141,His144,Leu140,Ala4,Glu119<br>Hydrogen Bonds: Thr6, Tyr120
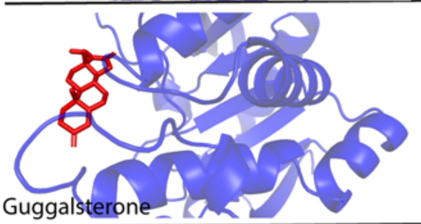 Guggalsterone	Hydrophobic Bonds: Phe212,Gly211,Tyr213,Phe214<br>Hydrogen Bonds: Thr91, Thr94
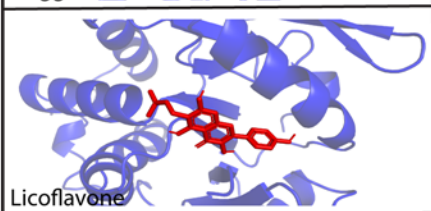 Licoflavone	Hydrophobic Bonds: Asp61,Arg137,Lys5,Leu140,Val118,His144,Arg191<br>Hydrogen Bonds: Lys117,Glu119,Ala4,Thr6
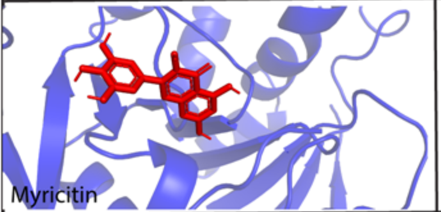 Myricitin	Hydrophobic Bonds: His293,Ala233,Ala234,Glu165,Thr158<br>Hydrogen Bonds: Val237,Arg154,Ile157,Lys161
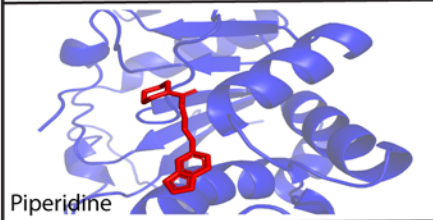 Piperidine	Hydrophobic Bonds: Val183,Tyr213,Phe212,Ser210,Gly211,Cys90,Thr91,Ser209<br>Hydrogen Bonds: Phe214
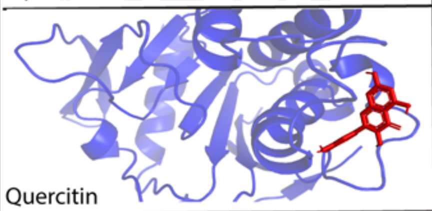 Quercitin	Hydrophobic Bonds: Ala99,Ile72,Asp75,Arg96,Tyr78,Lys95,Ser83<br>Hydrogen Bonds: Glu92,Phe82
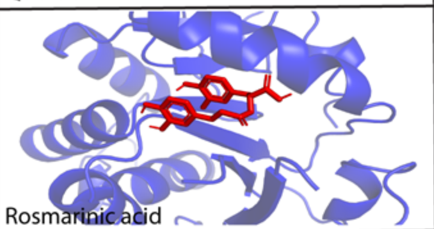 Rosmarinic acid	Hydrophobic Bonds: Lys141,Tyr120,Ala4,His144,Leu140<br>Hydrogen Bonds: Thr6,Glu119,Asp61,Arg137
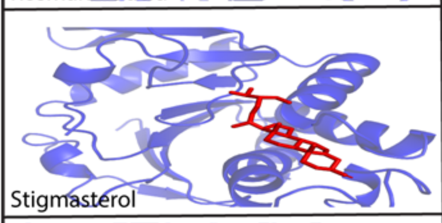 Stigmasterol	Hydrophobic Bonds: Cys90,Thr94,Ser209,Ser210,Phe212,Gly211,Phe214,Tyr213<br>Hydrogen Bonds: Asn87,Met89
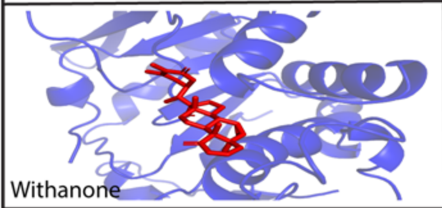 Withanone	Hydrophobic Bonds: Cys99,Gly211,Phe212,Tyr213,Phe214<br>Hydrogen Bonds: Thr94,Thr91

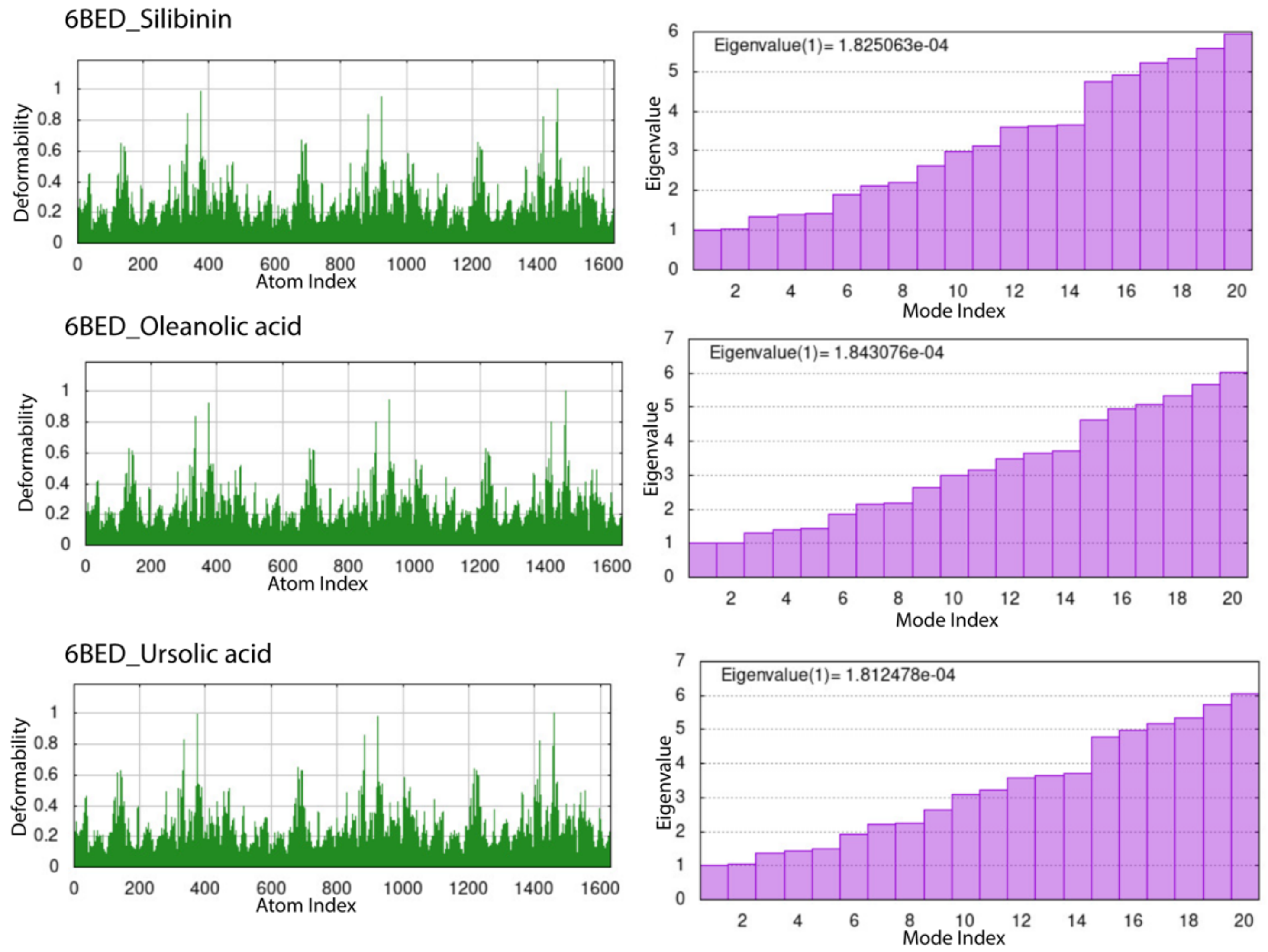

6BED_Silibinin
Deformability
Atom Index
Eigenvalue(1)= 1.825063e-04
Eigenvalue
Mode Index
6BED_Oleanolic acid
Deformability
Atom Index
Eigenvalue(1)= 1.843076e-04
Eigenvalue
Mode Index
6BED_Ursolic acid
Deformability
Atom Index
Eigenvalue(1)= 1.812478e-04
Eigenvalue
Mode Index

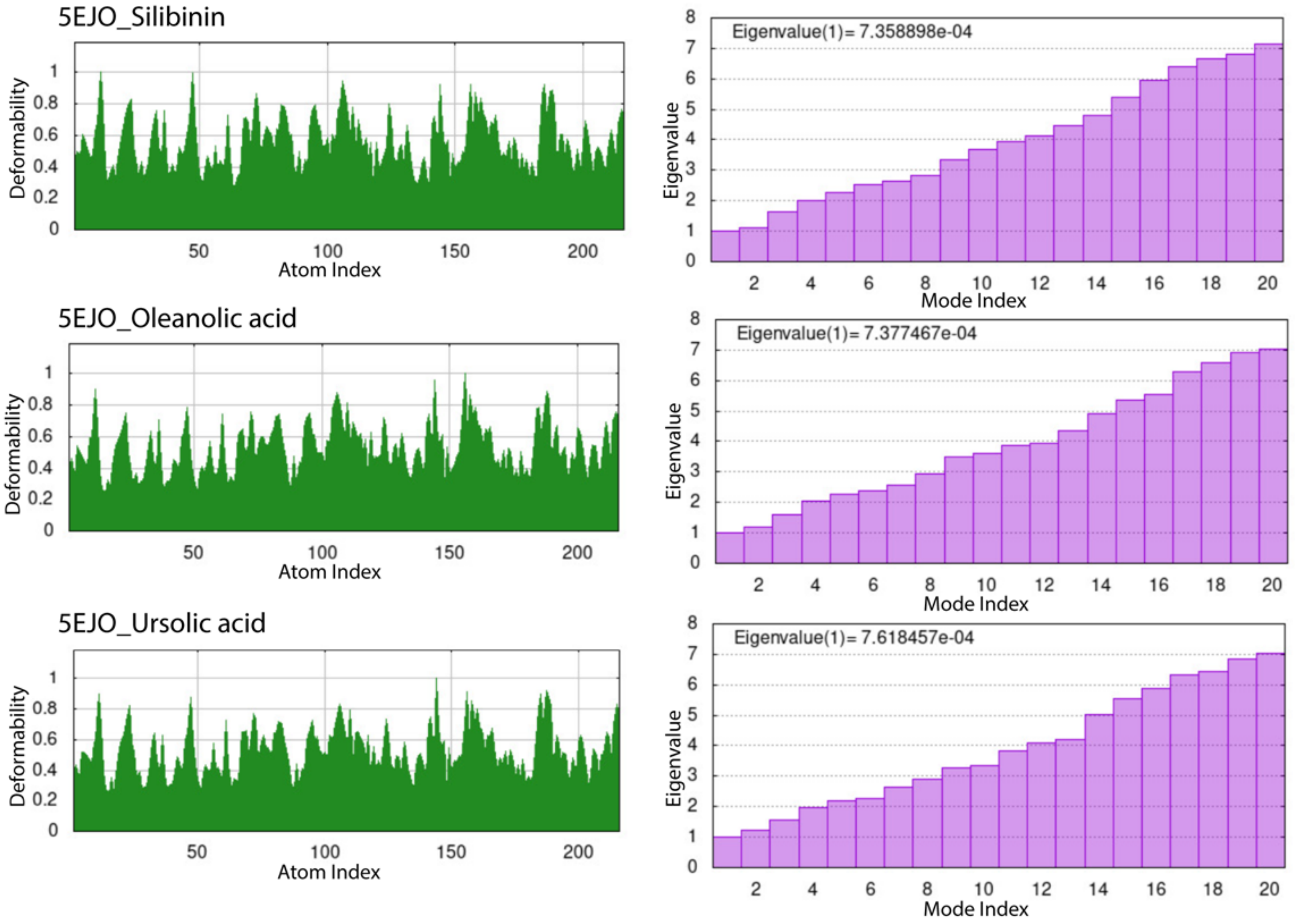
5EJO_Silibinin
Deformability
Atom Index
Eigenvalue(1)= 7.358898e-04
Eigenvalue
Mode Index
5EJO_Oleanolic acid
Deformability
Atom Index
Eigenvalue(1)= 7.377467e-04
Eigenvalue
Mode Index
5EJO_Ursolic acid
Deformability
Atom Index
Eigenvalue(1)= 7.618457e-04
Eigenvalue
Mode Index

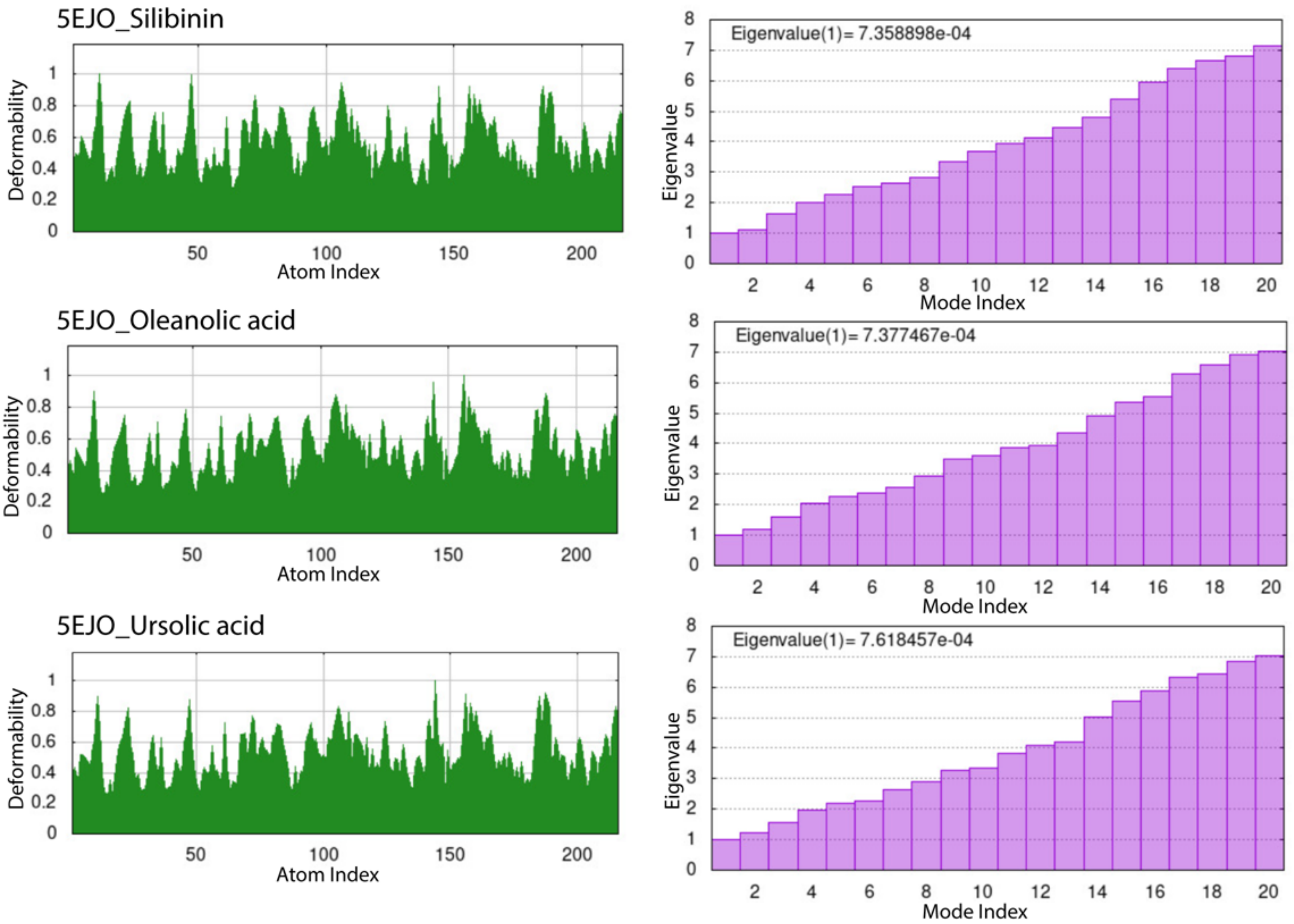
5EJO_Silibinin
Deformability
Atom Index
Eigenvalue(1)= 7.358898e-04
Eigenvalue
Mode Index
5EJO_Oleanolic acid
Deformability
Atom Index
Eigenvalue(1)= 7.377467e-04
Eigenvalue
Mode Index
5EJO_Ursolic acid
Deformability
Atom Index
Eigenvalue(1)= 7.618457e-04
Eigenvalue
Mode Index

6BED_Silibinin

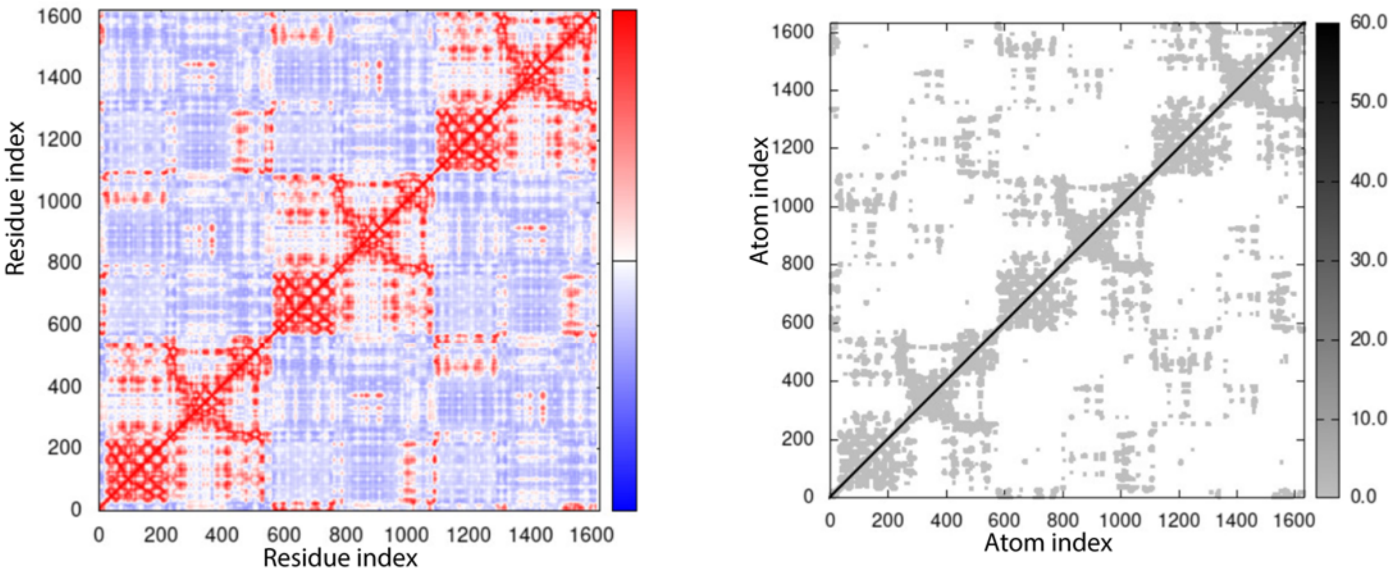

6BED_Oleanolic acid

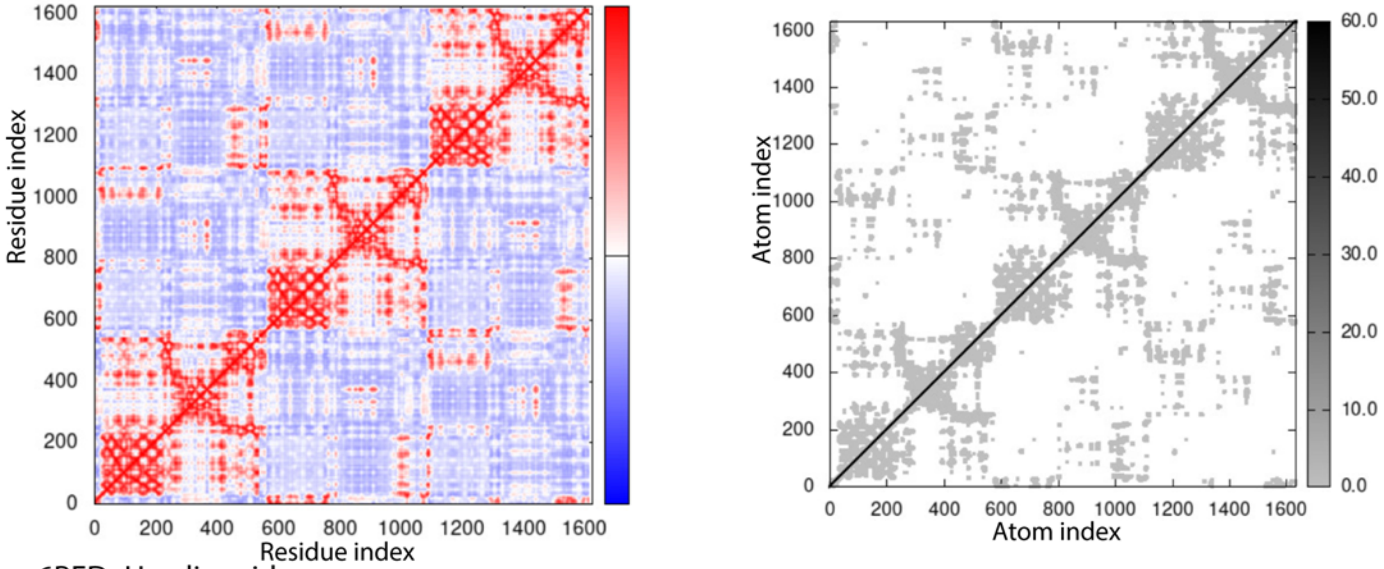

6BED_Ursolic acid

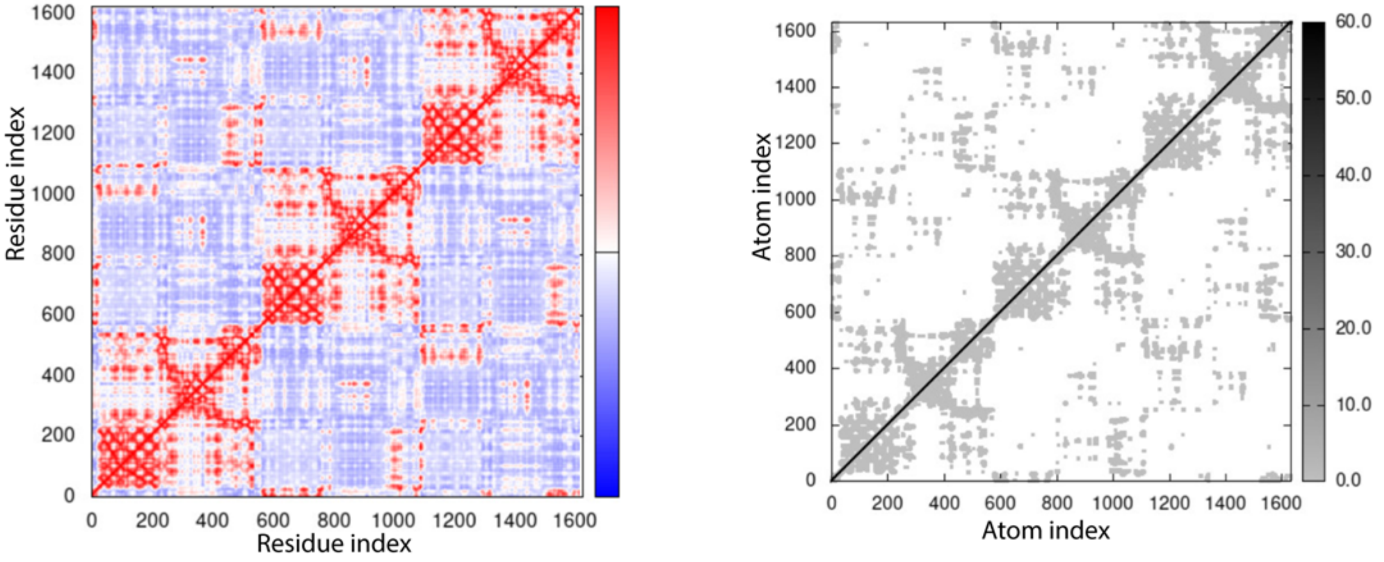

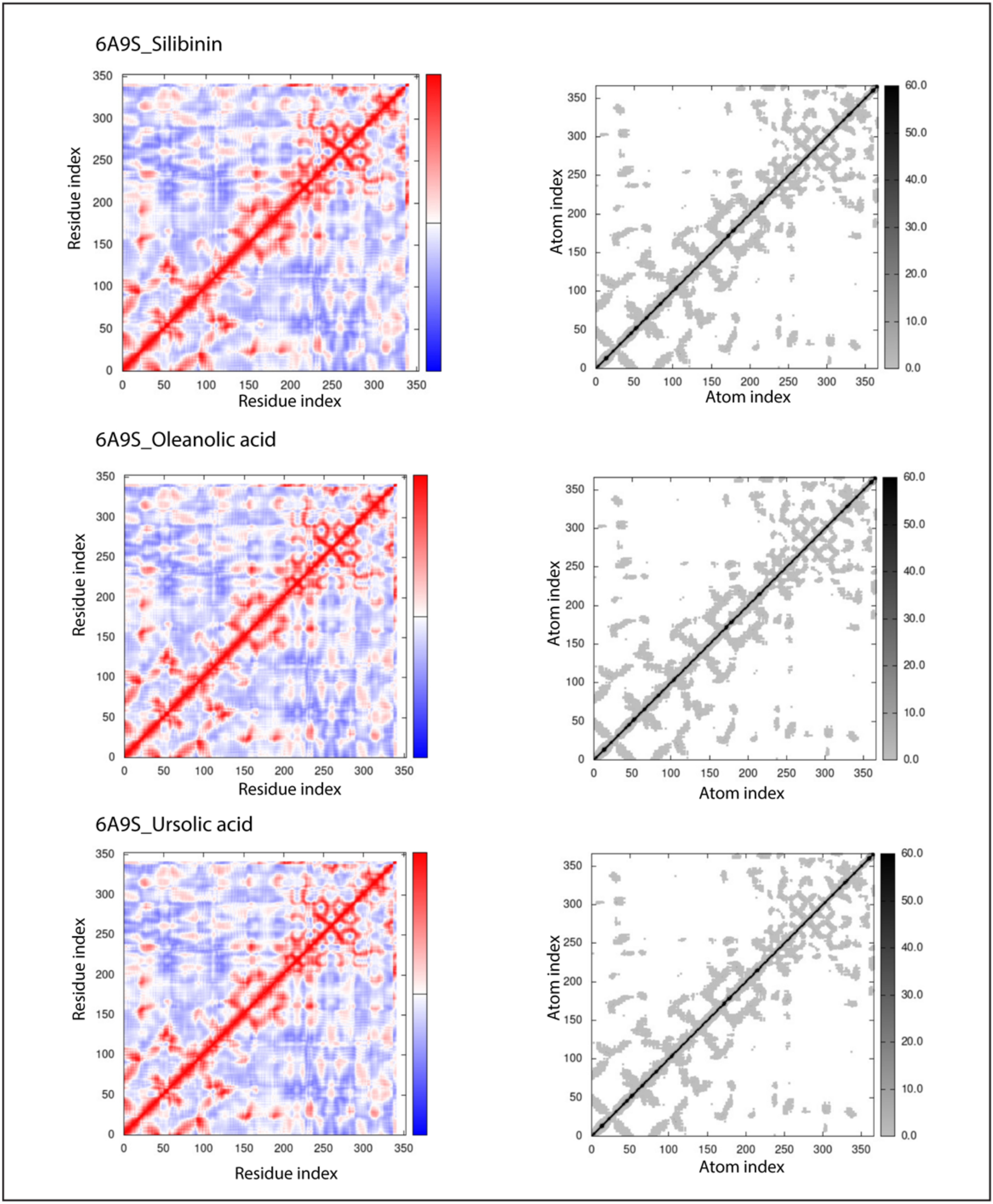
6A9S_Silibinin
Residue index
Residue index
Atom index
Atom index
0
50
100
150
200
250
300
350
0.0
10.0
20.0
30.0
40.0
50.0
60.0
6A9S_Oleanolic acid
Residue index
Residue index
Atom index
Atom index
6A9S_Ursolic acid
Residue index
Residue index
Atom index
Atom index

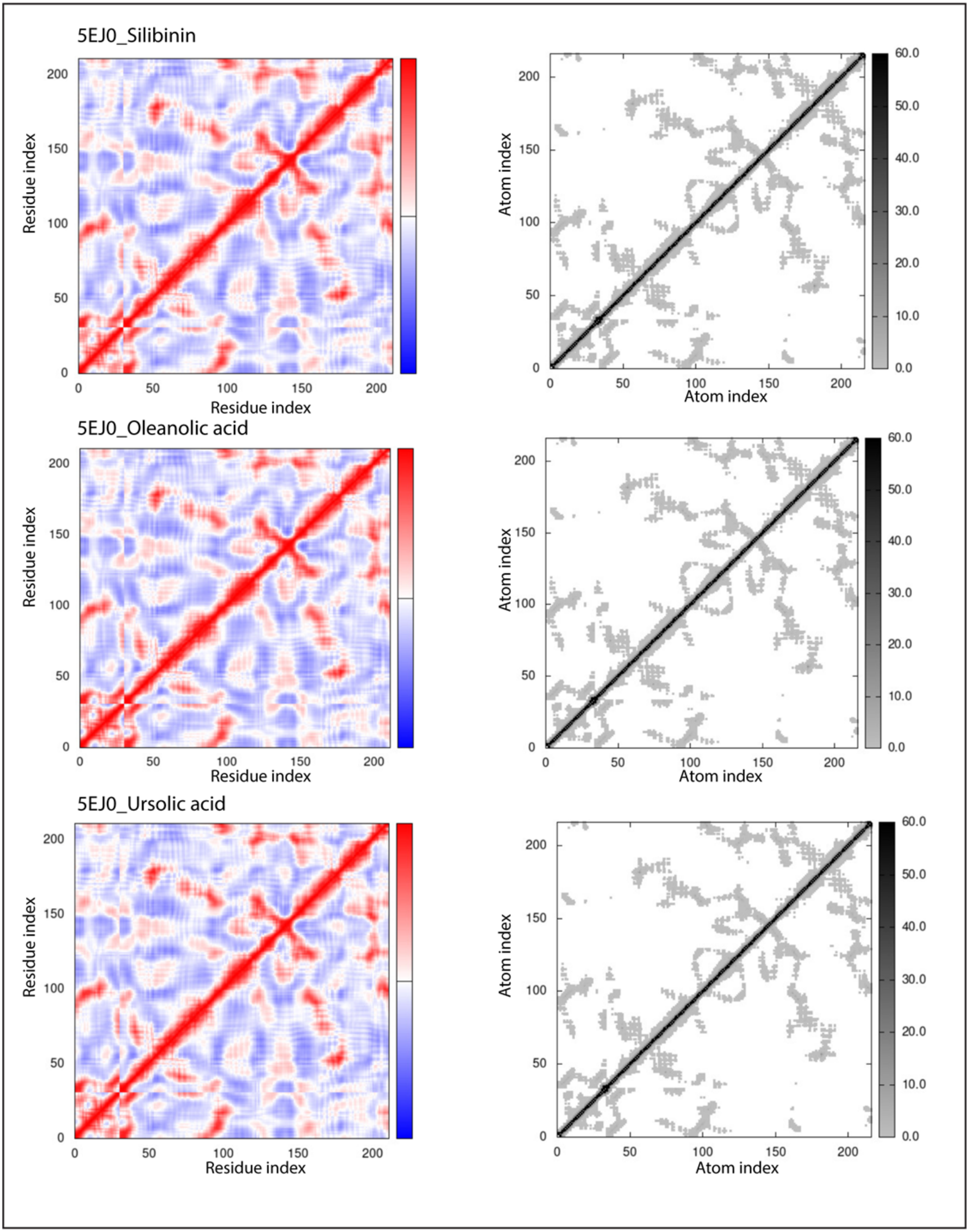
5EJ0_Silibinin
Residue index
Residue index
Atom index
Atom index
60.0
50.0
40.0
30.0
20.0
10.0
0.0
5EJ0_Oleanolic acid
Residue index
Residue index
Atom index
Atom index
5EJ0_Ursolic acid
Residue index
Residue index
Atom index
Atom index
