# Supplementary material for: Targeting envelope proteins of poxviruses to repurpose phytochemicals against monkeypox: An in silico investigation
Source: Front Microbiol. 2023 Jan 5;13:1073419. doi: 10.3389/fmicb.2022.1073419 (PMC9849581; doi:10.3389/fmicb.2022.1073419)
Supplement: Supplementary file 2 [file Table_1.DOC]

**Supplementary Tables**

| **Pharmacokinetic property** | **Model name** | **Computationally predicted values for the phytochemicals (ADMET profile)** | | | | | | **Measurement units** |
| --- | --- | --- | --- | --- | --- | --- | --- | --- |
|  |  | **Curcumin** | **E-Guggulsterone** | **Licoflavone A** | **Myricetin** | **Oleanolic acid** | **Piperidine** |  |
| Absorption | Water solubility | -4.01 | -4.931 | -3.474 | -2.915 | -3.074 | -0.156 | Numeric (log mol/L) |
|  | Caco2 permeability | -0.093 | 1.294 | 0.934 | 0.095 | 1.17 | 1.347 | Numeric (log Papp in 10^-6^ cm/s) |
|  | Intestinal absorption (human) | 82.19 | 99.655 | 91.625 | 65.93 | 99.931 | 100 | Numeric (% Absorbed) |
|  | Skin Permeability | -2.764 | -2.465 | -2.736 | -2.735 | -2.735 | -2.795 | Numeric (log K_p_) |
|  | P-glycoprotein substrate | Yes | No | Yes | Yes | No | Yes | Categorical (Yes/No) |
|  | P-glycoprotein I inhibitor | Yes | Yes | No | No | No | No | Categorical (Yes/No) |
|  | P-glycoprotein II inhibitor | Yes | No | No | No | No | No | Categorical (Yes/No) |
| Distribution | VDss (human) | -0.215 | 0.158 | 0.867 | 1.317 | -1.085 | 0.503 | Numeric (log L/kg) |
|  | Fraction unbound (human) | 0 | 0.001 | 0.037 | 0.238 | 0 | 0.808 | Numeric (Fu) |
|  | BBB permeability | -0.562 | 0.141 | -0.57 | -1.493 | -0.14 | 0.024 | Numeric (log BB) |
|  | CNS permeability | -2.99 | -2.02 | -1.957 | -3.709 | -1.157 | -2.829 | Numeric (log PS) |
| Metabolism | CYP2D6 substrate | No | No | No | No | No | No | Categorical (Yes/No) |
|  | CYP3A4 substrate | Yes | Yes | No | No | Yes | No | Categorical (Yes/No) |
|  | CYP1A2 inhibitor | Yes | No | No | Yes | No | No | Categorical (Yes/No) |
|  | CYP2C19 inhibitor | Yes | No | No | No | No | No | Categorical (Yes/No) |
|  | CYP2C9 inhibitor | Yes | No | No | No | No | No | Categorical (Yes/No) |
|  | CYP2D6 inhibitor | No | No | No | No | No | No | Categorical (Yes/No) |
|  | CYP3A4 inhibitor | Yes | Yes | No | No | No | No | Categorical (Yes/No) |
| Excretion | Total Clearance | -0.002 | 0.61 | 0.086 | 0.422 | -0.081 | 1.093 | Numeric (log ml/min/kg) |
|  | Renal OCT2 substrate | No | No | No | No | No | No | Categorical (Yes/No) |
| Toxicity | AMES toxicity | No | No | No | No | No | No | Categorical (Yes/No) |
|  | Maximum tolerated dose (human) | 0.081 | -0.455 | 0.332 | 0.51 | 0.203 | 0.964 | Numeric (log mg/kg/day) |
|  | hERG I inhibitor | No | No | No | No | No | No | Categorical (Yes/No) |
|  | hERG II inhibitor | No | Yes | No | No | No | No | Categorical (Yes/No) |
|  | Oral Rat Acute Toxicity (LD50) | 1.833 | 1.843 | 2.284 | 2.497 | 2.349 | 2.218 | Numeric (mol/kg) |
|  | Oral Rat Chronic Toxicity (LOAEL) | 2.228 | 1.769 | 2.611 | 2.718 | 2.085 | 1.466 | Numeric (log mg/kg-bw/day) |
|  | Hepatotoxicity | No | Yes | No | No | Yes | No | Categorical (Yes/No) |
|  | Skin Sensitization | No | No | No | No | No | No | Categorical (Yes/No) |
|  | *T. Pyriformis* toxicity | 0.494 | 0.808 | 0.397 | 0.286 | 0.285 | -0.78 | Numeric (log μg/L) |
|  | Minnow toxicity | -0.081 | 0.312 | 1.584 | 5.023 | -0.823 | 2.683 | Numeric (log mM) |

**Table S1.** Pharmacokinetic properties (ADMET: Absorption, Distribution, Metabolism, Excretion, Toxicity) of the test phytochemicals generated using the pkCSM - pharmacokinetics server.

| **Pharmacokinetic property** | **Model name** | **Computationally predicted values for the phytochemicals (ADMET profile)** | | | | | | **Measurement units** |
| --- | --- | --- | --- | --- | --- | --- | --- | --- |
|  |  | **Quercetin** | **Rosmarinic acid** | **Silibinin** | **Stigmasterol** | **Ursolic acid** | **Withanone** |  |
| Absorption | Water solubility | -2.925 | -3.059 | -3.204 | -6.682 | -3.072 | -5.127 | Numeric (log mol/L) |
|  | Caco2 permeability | -0.229 | -0.937 | 0.435 | 1.213 | 1.171 | 0.849 | Numeric (log Papp in 10^-6^ cm/s) |
|  | Intestinal absorption (human) | 77.207 | 32.516 | 61.861 | 94.97 | 100 | 100 | Numeric (% Absorbed) |
|  | Skin Permeability | -2.747 | -2.735 | -2.78 | -2.783 | -2.735 | -3.365 | Numeric (log K_p_) |
|  | P-glycoprotein substrate | Yes | Yes | Yes | No | No | Yes | Categorical (Yes/No) |
|  | P-glycoprotein I inhibitor | No | No | Yes | Yes | No | Yes | Categorical (Yes/No) |
|  | P-glycoprotein II inhibitor | No | No | Yes | Yes | No | Yes | Categorical (Yes/No) |
| Distribution | VDss (human) | 1.559 | 0.393 | 0.369 | 0.178 | -1.088 | 0.081 | Numeric (log L/kg) |
|  | Fraction unbound (human) | 0.206 | 0.348 | 0 | 0 | 0 | 0.154 | Numeric (Fu) |
|  | BBB permeability | -1.098 | -1.378 | -1.207 | 0.771 | -0.141 | -0.259 | Numeric (log BB) |
|  | CNS permeability | -3.065 | -3.347 | -3.639 | -1.652 | -1.187 | -2.719 | Numeric (log PS) |
| Metabolism | CYP2D6 substrate | No | No | No | No | No | No | Categorical (Yes/No) |
|  | CYP3A4 substrate | No | No | No | Yes | Yes | Yes | Categorical (Yes/No) |
|  | CYP1A2 inhibitor | Yes | No | No | No | No | No | Categorical (Yes/No) |
|  | CYP2C19 inhibitor | No | No | No | No | No | No | Categorical (Yes/No) |
|  | CYP2C9 inhibitor | No | No | Yes | No | No | No | Categorical (Yes/No) |
|  | CYP2D6 inhibitor | No | No | No | No | No | No | Categorical (Yes/No) |
|  | CYP3A4 inhibitor | No | No | No | No | No | No | Categorical (Yes/No) |
| Excretion | Total Clearance | 0.407 | 0.25 | -0.103 | 0.618 | 0.083 | 0.385 | Numeric (log ml/min/kg) |
|  | Renal OCT2 substrate | No | No | No | No | No | Yes | Categorical (Yes/No) |
| Toxicity | AMES toxicity | No | No | No | No | No | No | Categorical (Yes/No) |
|  | Maximum tolerated dose (human) | 0.499 | 2.811 | 0.65 | 0.664 | 0.199 | -1.093 | Numeric (log mg/kg/day) |
|  | hERG I inhibitor | No | No | No | No | No | No | Categorical (Yes/No) |
|  | hERG II inhibitor | No | No | Yes | Yes | No | No | Categorical (Yes/No) |
|  | Oral Rat Acute Toxicity (LD50) | 2.471 | 2.811 | 2.559 | 2.54 | 2.346 | 2.907 | Numeric (mol/kg) |
|  | Oral Rat Chronic Toxicity (LOAEL) | 2.612 | 2.907 | 3.494 | 0.872 | 2.054 | 1.676 | Numeric (log mg/kg-bw/day) |
|  | Hepatotoxicity | No | No | No | No | Yes | No | Categorical (Yes/No) |
|  | Skin Sensitization | No | No | No | No | No | No | Categorical (Yes/No) |
|  | *T. Pyriformis* toxicity | 0.288 | 0.302 | 0.285 | 0.433 | 0.285 | 0.299 | Numeric (log μg/L) |
|  | Minnow toxicity | 3.721 | 2.698 | 2.543 | -1.675 | -0.787 | 1.322 | Numeric (log mM) |

**Table S2.** Pharmacokinetic properties (ADMET: Absorption, Distribution, Metabolism, Excretion, Toxicity) of the test phytochemicals generated using the pkCSM - pharmacokinetics server.

| **S.No.** | **Name of the phytochemical** | **Binding Free Energy (in kcal/mol)** | **Interacting amino acids on the D13 protein** | **# Hydrogen bonds** |
| --- | --- | --- | --- | --- |
| 1. | Curcumin | -7.7 | Phe481, Leu467, Thr478, Asn480, Thr468, Ser256, Ser254, Tyr258, Asp166, Asn464, Thr474, Glu114, Asn117 | 5 |
| 2. | E-Guggulsterone | -8.6 | Val315, Val330, Pro316, Ile332, Ala375, Asn334, Asp333, Asp374, Val335, Ser350, Val341 | 1 |
| 3. | Licoflavone | -8.2 | Asn480, Ser254, Val528, Ser256, Tyr258, Thr478, Glu230, Lys484, Asn530, Glu114 | 5 |
| 4. | Myricitrin | -8.4 | Gly473, Asn464, Ile120, Asn121, Asn117, Thr474, Thr476, Ser256, Glu230, Lys484, Asn530, Asn118, Ser470 | 8 |
| 5. | Oleanolic acid | -9.3 | Glu280, Lys434, Lys436, Ile462, Arg461, Tyr155, Lys127, Val154, His128, Asn472 | 3 |
| 6. | Piperidine | -8.8 | Asn435, Lys429, Asn472, Ile120, Gly473, Asn117, Thr468, Ser470 | 2 |
| 7. | Quercetin | -9.0 | Pro88, Tyr89, Tyr116, Phe433, Ile428, Phe427, Asn435, Asn117, Gln278, Lys429 | 3 |
| 8. | Rosmarinic acid | -8.9 | Thr474, Asn117, Ileu428, Gly473, Ile120, Asn435, Phe427, Lys434, Phe433, Lys429, Lys93, Asn472, Ile120, Tyr89, Tyr116, Asp425, Asn472 | 4 |
| 9. | Silibinin | -9.2 | Lys127, Ser152, Thr153, Ile440, Ser441, Asn456, Arg461, Glu465, Glu74, His128, Asp438, Gly457, Pro458, Lys127, Ser152, Thr153, Ile440, Ser441, Asn456, Arg461, Glu465 | 10 |
| 10. | Stigmasterol | -7.8 | Asp166, Asn480, Phe481, Lys484, Asn464, Thr478, Thr474, Asn472, Asn117, Ser250, Asn118 | 4 |
| 11. | Ursolic acid | -9.2 | His128, Thr153, Lys127, Val134, Tyr155, Glu280, Lys434, Lys436, Arg461, Ileu462 |  |
| 12. | Withanone | -8.6 | Lys127, Glu465, Gly457, Asn456, Pro458, Thr153, Tyr155, Ser152, Arg461 | 4 |
| 13. | Rifampicin (Control) | -9.8 | Asp285, His386, Ser385, Asn389, Thr147, Ile148, Glu150, Glu134, Ser387, Asp137, Ser366 | 1 |

**Table S3**. Amino acid residues on the D13 protein of poxvirus found to be interacting with the test phytochemicals.

| **S.No.** | **Name of the phytochemical** | **Binding Free Energy (in kcal/mol)** | **Interacting amino acids on the A26 protein** | **# Hydrogen bonds** |
| --- | --- | --- | --- | --- |
| 1. | Curcumin | -7.7 | Lys163, His167, Tyr160, Glu156, Asp136, Thr138, Asn134, Pro139, Arg157 | 4 |
| 2. | E-Guggulsterone | -7.5 | Thr283, Lys311, Asn340, Asp310, Asn284, Asn285, Ile337  Tyr289, Arg46 | 2 |
| 3. | Licoflavone | -7.4 | Thr160, Arg157, Arg205, Ile202, His167, Glu156, Lys163, Asp201 | 3 |
| 4. | Myricitrin | -7.3 | Ile337, Asn340, Thr283, Lys311, Asp339, Tyr289, Arg46, Asp310 | 4 |
| 5. | Oleanolic acid | -7.9 | Tyr242, Asp157, Glu54, Arg57, Asp58, Lys61, Lys306, Thr303, Asp267 | 2 |
| 6. | Piperidine | -7.1 | His135, Tyr113, Asp136, Tyr160, Gln164, Ile202, Lys163,  Asp201, Thr303 | 0 |
| 7. | Quercetin | -7.3 | Asn340, Ile337, Thr283, Lys311, Tyr289, Asp339, Ile338,  Arg46, Asp310 | 5 |
| 8. | Rosmarinic acid | -6.2 | Thr109, Asp136, Leu110, Asn111, Gly137, Tyr124, Phe107  Lys108, His135, Lys133 | 3 |
| 9. | Silibinin | -7.8 | Asn334, Thr353, Asp339, Glu341, Asn287, Asn284, Arg46, Thr283, Asp310, Asn340, Arg333, Thr355 | 3 |
| 10. | Stigmasterol | -8.0 | Ile202, His167, Lys163, Tyr160, Gln164, His135, Tyr113, Asp136 | 0 |
| 11. | Ursolic acid | -7.8 | Lys61, Lys306, Lys301, Glu304, Val302, Thr303, Arg57, Asp267, Phe266 | 3 |
| 12. | Withanone | -7.8 | Glu341, Thr283, Asp339, Asp310, Lys311, Asn287, Tyr289, Asn340 | 3 |
| 13. | Rifampicin (Control) | -8.0 | Tyr113, Tyr160, Gln164, Arg205, Asp201, Ile202, His167, Lys163 | 2 |

**Table S4**. Amino acid residues on the A26 protein of poxvirus found to be interacting with the test phytochemicals.

| **S.No.** | **Name of the phytochemical** | **Binding Free Energy (in kcal/mol)** | **Interacting amino acids on the A26 protein** | **# Hydrogen bonds** |
| --- | --- | --- | --- | --- |
| 1. | Curcumin | -6.6 | Tyr62, Arg137, Lys141, His144, Leu140, Ala4, Glu119  Thr6, Tyr120 | 2 |
| 2. | E-Guggulsterone | -7.1 | Phe212, Gly211, Tyr213, Phe214, Thr91, Thr94 | 2 |
| 3. | Licoflavone | -6.9 | Asp61, Arg137, Lys5, Leu140, Val118, His144, Arg191  Lys117, Glu119, Ala4, Thr6 | 4 |
| 4. | Myricitrin | -6.9 | His293, Ala233, Ala234, Glu165, Thr158, Val237, Arg154, Ile157, Lys161 | 4 |
| 5. | Oleanolic acid | -7.8 | Tyr181, Ile156, Asp182, Thr94, Ser210, Cys90, Phe212, Gly211, Ser209, Tyr215, Phe214, Val183 | 1 |
| 6. | Piperidine | -7.3 | Val183, Tyr213, Phe212, Ser210, Gly211, Cys90, Thr91, Ser209  Phe214 | 1 |
| 7. | Quercetin | -7.2 | Ala99, Ile72, Asp75, Arg96, Tyr78, Lys95, Ser83, Glu92, Phe82 | 2 |
| 8. | Rosmarinic acid | -6 | Lys141, Tyr120, Ala4, His144, Leu140, Thr6, Glu119, Asp61, Arg137 | 4 |
| 9. | Silibinin | -7.5 | Ser184, Arg154, Tyr215, Ile156, Asp182,Tyr184, Gly211, Phe212, Cys90, Thr94, Val183 | 2 |
| 10. | Stigmasterol | -7.5 | Cys90, Thr94, Ser209, Ser210, Phe212, Gly211, Phe214, Tyr213  Asn87, Met89 | 2 |
| 11. | Ursolic acid | -8.0 | Val183, Phe212, Gly211, Cys90, Tyr213, Phe214, Asp182, Tyr181 | 1 |
| 12. | Withanone | -7.7 | Cys99, Gly211, Phe212, Tyr213, Phe214, Thr94, Thr91 | 2 |
| 13. | Rifampicin (Control) | -7.7 | Phe216, Tyr213, Phe212, Thr91, Ser216, Cys86, Cys90, Gly211, Asn87 | 2 |

**Table S5**. Amino acid residues on the H3 protein of poxvirus found to be interacting with the test phytochemicals.

**Table S6**. Predicted cellular targets and their ligand similarity scores with Ursolic acid.

**Table S7**. Predicted cellular targets and their ligand similarity scores with Oleanolic acid.

**Table S8**. Predicted cellular targets and their ligand similarity scores with Silibinin.

| **Name of Phytochemical** | **Inhibition constant (K*_i_*) (in μM) against viral proteins** | | |
| --- | --- | --- | --- |
|  | **D13** | **A26** | **H3** |
| Ursolic acid | 15.14 | 13.18 | 13.52 |
| Oleanolic acid | 15.71 | 13.35 | 13.18 |
| Silibinin | 15.54 | 13.18 | 12.67 |
| Rifampicin (Control) | 16.56 | 13.52 | 13.01 |

**Table S9**. Inhibition potential of the lead phytochemicals and rifampicin (control) against various envelope proteins of monkeypox.
